# Poly(A) inclusive RNA isoform sequencing (PAIso−seq) reveals wide-spread non-adenosine residues within RNA poly(A) tails

**DOI:** 10.1038/s41467-019-13228-9

**Published:** 2019-11-22

**Authors:** Yusheng Liu, Hu Nie, Hongxiang Liu, Falong Lu

**Affiliations:** 1 State Key Laboratory of Molecular Developmental Biology, Institute of Genetics and Developmental Biology, Chinese Academy of Sciences, 100101 Beijing, China; 2 University of Chinese Academy of Sciences, 100049 Beijing, China; 3 The Innovative Academy of Seed Design, Chinese Academy of Sciences, 100101 Beijing, China

**Keywords:** RNA sequencing, RNA sequencing, RNA modification

## Abstract

Message RNA poly(A) tails are vital for their function and regulation. However, the full-length sequence of mRNA isoforms with their poly(A) tails remains undetermined. Here, we develop a method at single-cell level sensitivity that enables quantification of poly(A) tails along with the full-length cDNA while reading non-adenosine residues within poly(A) tails precisely, which we name poly(A) inclusive RNA isoform sequencing (PAIso−seq). Using this method, we can quantify isoform specific poly(A) tail length. More interestingly, we find that 17% of the mRNAs harbor non-A residues within the body of poly(A) tails in mouse GV oocytes. We show that PAIso−seq is sensitive enough to analyze single GV oocytes. These findings will not only provide an accurate and sensitive tool in studying poly(A) tails, but also open a door for the function and regulation of non-adenosine modifications within the body of poly(A) tails.

## Abstract (web summary)

The poly(A) tails on mRNA are vital for their function but it is difficult to map full-length sequences of mRNA isoforms with the entire poly(A) tails. Here the authors develop PAIso−seq which can measure isoform specific poly(A) tail length and base composition at single-cell sensitivity.

## Introduction

Mature RNAs, including messenger RNAs (mRNAs) and long noncoding RNAs (lncRNAs), are subjected to many post-transcriptional modifications. The 3′ end of most mRNAs and lncRNAs is cleaved co-transcriptionally with non-templated poly(A) tails added by poly(A) polymerases. The poly(A) tails have been known to be one of the key factors regulating RNA stability and translational efficiency^1–7^. Poly(A) + mRNAs and lncRNAs account for a small fraction of cellular RNA unless poly(A) + RNAs are enriched, whereas ribosomal RNAs (rRNAs) and transfer RNAs (tRNAs) are highly abundant. Oligo(dT)-based affinity purification is often used to enrich mRNA, but inevitably introduces bias toward poly(A) + RNAs with long poly(A) tails^8^. Therefore, it is better to avoid poly(A) enrichment to preserve the original poly(A) information when analyzing poly(A) tails.

The transcriptome has been extensively studied in the age of next-generation sequencing (NGS), with the exception of the detailed composition of poly(A) tails because the current NGS platforms cannot handle homopolymeric sequences longer than 30 nucleotides (nt) by using standard base-calling algorithm. Even for Sanger sequencing, long homopolymers are difficult to handle. Smart-seq2, one of the most sensitive single-cell RNA-sequencing (RNA-seq) technology, uses 3′-untranslated region (UTR) anchored oligo-dT primer (5′-AAGCAGTGGTATCAACGCAGAGTACT30VN-3′, where “N” is A, T, C, or G and “V” is A, C, or G) for reverse transcription to construct the complementary DNA (cDNA) library. The two terminal nucleotides “N” and “V” anchor the reverse transcriptase (RT) primer to the end of 3′-UTR and discard the poly(A) tails from the final cDNA library to avoid the homopolymeric sequences^9^. The other commonly used RNA-seq tools on Illumina platform also ignored poly(A) sequences during library preparation, sequencing, or data analysis steps, because it suffers from phasing errors and persisting signal from accumulation of uncleaved fluorescent molecules when sequencing homopolymers. The RNA isoform-sequencing (Iso-seq) on PacBio platform uses a strategy similar to Smart-seq2 to amplify cDNAs that discard poly(A) from the amplified cDNA. Therefore, regular RNA-seq or Iso-seq data from Illumina or PacBio platforms all lack poly(A) information.

Recently, there are two methods, PAL-seq (poly(A) tail length profiling by sequencing) and TAIL-seq, developed by using custom poly(T) (the reverse complement strand of poly(A) sequenced as poly(T) on Illumina platforms) base-calling algorithm to detect transcriptome-wide poly(A) tail length^1,8^. These methods have yielded rich knowledge of poly(A) tail length dynamics in germinal vesicle (GV) oocytes, spermatocytes, mouse embryonic fibroblast (MEFs), embryonic stem cells (ESCs), bone marrow, yeast, mouse liver, Arabidopsis leaves, Drosophila embryos, frog embryos, and zebrafish embryos^1,4,8,10–13^. PAL-seq utilizes a customized sequencing recipe that is only available on the discontinued Illumina Genome Analyzer II sequencer. The quantification of poly(A) tail length by using PAL-seq is based on proportional incorporation of biotin tag when doing primer extension with the mixture of dTTP and biotin-conjugated dUTP^1^. The chemistry underlying this method makes it unable to read non-adenosine residues within poly(A) tails as well as not at base resolution. TAIL-seq and its mRNA-enriched modified version, mTAIL-seq, are based on customized base-calling algorithm to determine the end of poly(T) sequences, which analyzes raw sequencing images available on limited sequencers. This is not accessible for most of the users relying on commercial sequencing service or standard sequencing facilities. This base-calling algorithm can determine the length of the poly(T) sequence up to 231 bp and can detect U, C, and G residues at 3′ termini of poly(A) tail^3,4,8^.

Transcription becomes silent in mouse oocytes before meiotic resumption. Therefore, many important developmental events in mouse oocyte maturation and early stage of embryo development, including maternal mRNA clearance and zygotic genome activation, highly depend on mRNA stored in GV oocyte that are known to be regulated at their poly(A) tails^10,14–17^. In this study, we develop a technology that can read transcriptome-wide full-length RNA isoforms with poly(A) tails accurately and sensitively by combination of extension after poly(A) tail to preserve the entire poly(A) sequence, template switching during full RNA isoform reverse transcription to sensitively amplify full-length cDNA with poly(A) tail, and third-generation sequencing on PacBio platform, which we named poly(A) inclusive RNA Isoform-sequencing (PAIso−seq). Templated end extension preserves the entire poly(A) sequence and provides a binding site for reverse transcription and PCR amplification. In addition, oligo-dT templated end extension only extends poly(A) + RNA that prevents the need of poly(A) + RNA enrichment. PacBio circular sequencing can accurately handle long homopolymers^18^. Moreover, circular sequencing makes a single molecule to be read multiple passes, which enables highly accurate generation of circular consensus sequences (CCSs or CCS reads).

By using this method, we comprehensively analyze the poly(A) tails in the transcriptome of mouse GV oocytes. We show that we can detect the length of poly(A) tails robustly even with single GV oocytes. Different isoforms transcribed from the same gene can have different poly(A) tails. In combination with previous mass-spectrometry data of mouse GV oocytes, we find that the high protein abundance genes were with significantly longer poly(A) tails, suggesting that longer poly(A) tails promote efficient translation of mRNAs in GV oocytes. Our method enables accurate base calling within poly(A) tails. To our great surprise, we see widespread non-adenosine residues within the body of many poly(A) tails besides the end of poly(A) tails. Therefore, PAIso−seq enables isoform-specific decomposition of RNA poly(A) tails, which reveals that the composition of poly(A) tails is far more complex than what we initially know, suggesting more mechanisms of poly(A) tail modification and regulation.

## Results

### PAIso−seq for accurate poly(A) tail analysis

Current methods in analyzing poly(A) tails on Illumina NGS platform are limited by the inability to handle long homopolymer sequences. TAIL-seq and PAL-seq used alternative poly(T) length calling algorithm or sequencing recipe to count the poly(T) length while sacrificing the ability to call non-A residues within RNA poly(A) tails, with the exception of the very 3′ end^1,8^. Moreover, they require microgram level of RNA input that is not feasible for rare in vivo or patient samples. The current development of PacBio third-generation sequencing enables reading of homopolymers through real-time single-molecule sequencing. In addition, looping of sequencing templates in sequencing libraries enables sequencing of a single template multiple passes to accurately call the consensus sequence read^19^. Therefore, PacBio third-generation sequencing platform may be the best choice to accurately analyze the length and composition of RNA poly(A) tails.

**Fig. 1:**
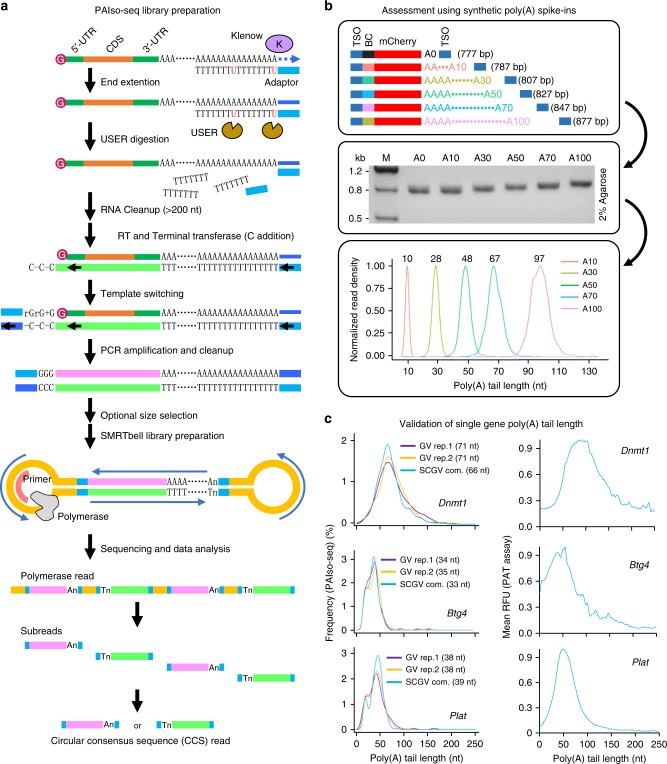
The principle and validation of PAIso−seq. **a** Flowchart for PAIso−seq method design. The main steps of the method include end-extension, template-switching, full-length cDNA amplification, circular adaptor ligation, and PacBio sequencing. **b** The structure (top panel) and agarose gel analysis of the poly(A) spike-ins (middle panel). The mean poly(A) tail length of each spike-in measured by PAIso−seq (bottom panel). Source data are provided as a Source Data file. **c** The poly(A) tail lengths of *Dnmt1*, *Btg4*, and *Plat* in GV oocytes measured by PAIso−seq (density plot of poly(A) tail length of detected CCS reads of the given genes, three replicates, left panel) and PAT assay by using capillary electrophoresis on fragment analyzer (mean of three replicates, right panel). The mean length of each gene poly(A) tail measured by PAIso−seq is shown. The number of CCS reads used are 141 (*Dnmt1/*GV rep.1), 249 (*Dnmt1/*GV rep.2), and 165 (*Dnmt1/*SCGV com.); 164 (*Btg4/*GV rep.1), 521 (*Btg4/*GV rep.2), and 357 (*Btg4/*SCGV com.); 136 (*Plat/*GV rep.1), 277 (*Plat/*GV rep.2), and 207 (*Plat/*SCGV com.). The average length of poly(A) tails are 74 nt (*Dnmt1*), 44 nt (*Btg4*), and 45 nt (*Plat*) measured by the PAT assay. RFU, relative fluorescence units.

We reason that if we can preserve the poly(A) information during reverse transcription, we will be able to analyze RNA poly(A) information precisely by using PacBio sequencing. To reduce the bias toward long poly(A) tails, we also want to avoid the poly(T) enrichment step. Therefore, we choose end extension of poly(A) + RNA with a guide primer containing adaptor sequence of template-switching oligo (TSO) sequence minus triple G bases at the 5′ end and with 5′-dUTTTTTTTdUTTTTTTT-3′ sequence at the 3′ end that can anneal to the end of poly(A) + RNAs (Fig. 1a and Supplementary Table 1). After end extension, the guide primer was removed by digestion with USER enzyme that cleaves at the two dU residues within the primer to avoid the guide primer as RT primer in the following reverse transcription step (Fig. 1a). Reverse transcription and template switching were performed in the presence of a primer corresponding to TSO sequence minus triple G bases and a TSO with the triple G at the end (Fig. 1a). Then, cDNA was amplified with single TSO oligo minus triple G to generate enough amount of cDNA ready for SMRTbell adaptor ligation (Fig. 1a). After adaptor ligation, the circular full-length cDNA library with poly(A) tails was sequenced on the PacBio platform to generate long polymerase reads up to 45 kb, which can have up to 200 passes of a single molecule to accurately call the CCS read representing a single full-length cDNA sequence including the poly(A) tails, which has been sequenced multiple times. The number of passes of a CCS read represent how many times the single cDNA sequence has been sequenced (Fig. 1a). Templated end extension and reverse transcription coupled with template switching are both highly efficient; therefore, this method can be very sensitive.

By using this method, we sequenced two full-length poly(A) + cDNA libraries from two independent biological replicates of mouse GV oocyte samples by using PAIso−seq. Each mappable CCS read is considered as a transcript detected. After mapping the CCSs to the mouse genome (GRCm38 build), the first library contains 79,994 transcripts, while the other library contains 227,902 transcripts (Supplementary Fig. 1a). In addition, we tested whether the method can be used for single-cell analysis by sequencing 15 single GV oocytes (see below for details). We combined all the data for the single cells as a third biological replicate containing 191,023 transcripts in total (Supplementary Fig. 1a). When combining all three replicates together, the GV oocyte dataset covers 11,538 genes with at least one transcript, and 8281 genes with at least three transcripts (Supplementary Fig. 1b).

To test whether PAIso−seq can assess the length of the poly(A) tails accurately, we spike in a pool of barcoded synthetic cDNAs with defined poly(A) tail lengths of 10, 30, 50, 70, and 100 nt, respectively, to the reverse-transcribed cDNA sample (Supplementary Fig. 2a). After sequencing, we observed the mean tail length of 10, 28, 48, 67, and 97 nt, which is very close to the expected length (Fig. 1b), demonstrating that our method can assess the poly(A) tail length accurately. For the GV oocyte sample, we can see that *Dnmt1* has relatively long poly(A) tails, while *Btg4* and *Plat* has relatively short poly(A) tails, which is generally consistent with the poly(A) length test (PAT) assay (see the “Methods” section) results for these genes (Fig. 1c). Therefore, poly(A) tail length from PAIso−seq can be verified by both spike-in standards and a sequencing-independent method, confirming the capability of this method in assessing poly(A) tail length.

### PAIso−seq has good reproducibility

**Fig. 2:**
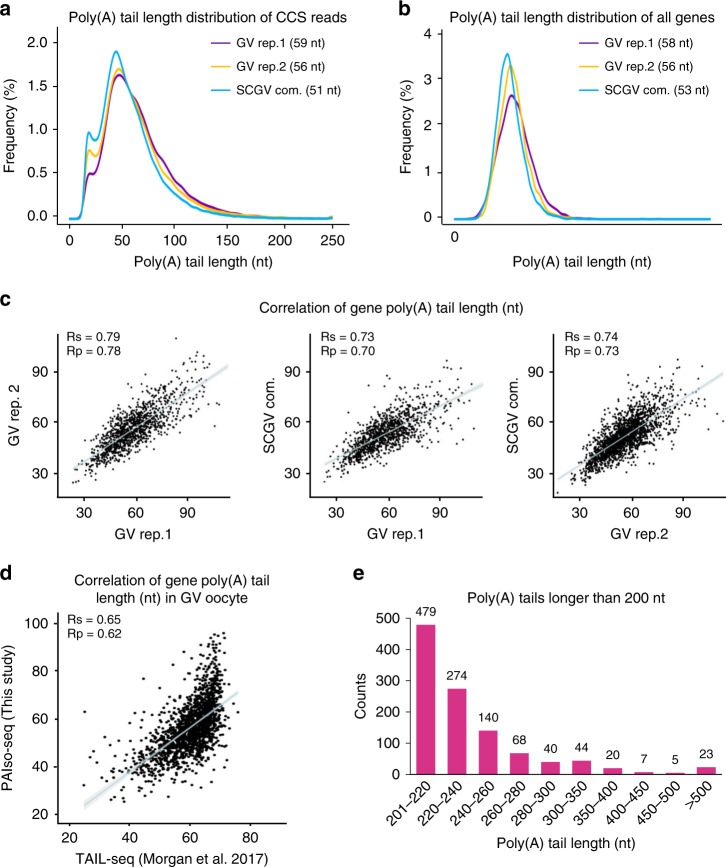
PAIso−seq captures poly(A) inclusive transcripts. **a** Global distribution of poly(A) tail lengths of all transcripts (CCSs) in GV oocytes. The median poly(A) tail length of CCS reads for each replicate is shown. **b** The distribution of poly(A) tail lengths of all genes. The median of mean poly(A) tail length of the genes for each replicate (with no less than three CCSs) is shown. **c** The correlation of gene (with no less than ten CCSs in each of the replicates) poly(A) tail length among three replicates of GV oocytes. The blue line represents linear regression line. The light-gray area represents confidence interval of the regression. *n* = 1179 (left panel), *n* = 1120 (middle panel), and *n* = 1992 (right panel). *R*_p_ and *R*_s_ refer to Pearson’s and Spearman’s correlation coefficient. **d** The correlation between gene poly(A) tail length in GV oocytes measured by PAIso−seq (this study) and TAIL-seq^10^. The blue line represents linear regression line. The light-gray area represents confidence interval of the regression. Genes with at least 10 reads in PAIso−seq and 30 tags in TAIL-seq datasets are included in the analysis (*n* = 1662). *R*_p_ and *R*_s_ refer to Pearson’s and Spearman’s correlation coefficient. **e** The distribution of poly(A) tails longer than 200 nt. The number above the bar shows the counts of CCS reads with given poly(A) tail length.

To analyze the reproducibility of PAIso−seq, we first compared whether we can capture the transcriptome well. We can see that the normalized read counts per gene show good correlation between each replicate (Supplementary Fig. 3). The global distribution patterns of poly(A) tail length per transcript and per gene are similar between each of the replicates (Fig. 2a, b). Moreover, the poly(A) tail length for each gene is highly reproducible between replicates (Fig. 2c). The poly(A) tail length in GV oocytes has been previously mapped by using TAIL-seq with maximum detection limit of 79 nt^10^. We can see that there is a good correlation between the poly(A) tail length determined by TAIL-seq and PAIso−seq (Fig. 2d), further confirming the performance of PAIso−seq in determining the poly(A) tail length. One obvious feature of PAIso−seq is that it has no obvious upper-size limit for detection. Poly(A) tails are commonly considered as no more than 250 nt in length, at which the enzyme can no longer bind to CPSF (cleavage and polyadenylation specificity factor) and polyadenylation stops^20,21^. When we look into individual poly(A) tails, we saw about 0.4% (1,100/297,868) of poly(A) tails longer than 200 nt and 0.1% (207/297,868) longer than 260 nt. Although the number of transcripts with long tails are small, they are very likely real, because for some of the genes, we can consistently detect transcripts with long tails from three independent replicates (Supplementary Table 4). In the future, it will be interesting to analyze the function and regulation of these long poly(A) tails that are not identifiable by previous methods.

### Isoform-specific polyadenylation

**Fig. 3:**
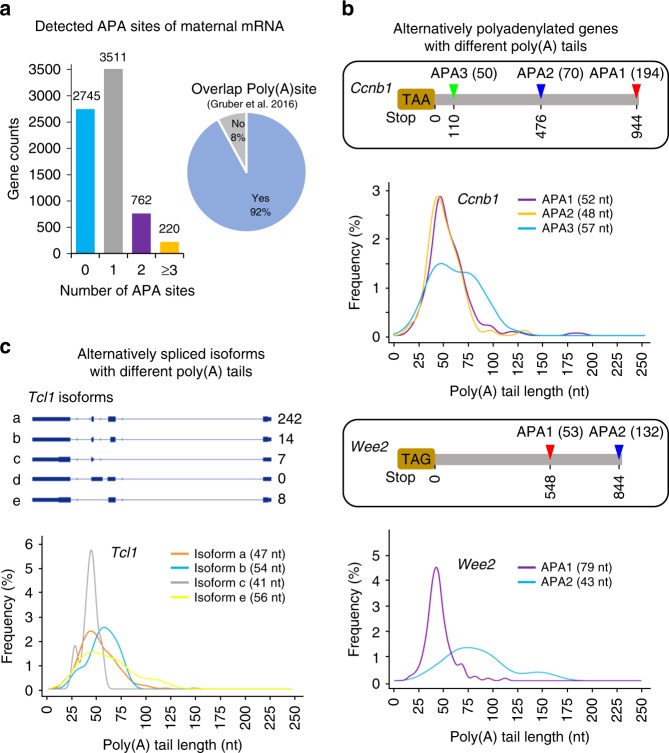
PAIso−seq enables detection of isoform-specific poly(A) tails. **a** Alternative polyadenylation (APA) events (left panel) of maternal transcripts in GV oocytes detected by PAIso−seq and compared with reference poly(A) site (right panel)^45^. **b** Two maternal genes, *Ccnb1* (with three polyadenylation sites, *p* = 0.0067 between APA2 and APA3) and *Wee2* (with two polyadenylation sites, *p* = 3.9e − 12 between two APA) with APA isoform-specific poly(A) tails. The number of APA isoforms are shown at the right of APA model. The mean length of poly(A) tails from isoforms of different polyadenylation sites is shown at the graph. The *p* value was calculated by unpaired and two-sided Student’s *t* test. The number of CCS reads used are 194 (*Ccnb1/*APA1), 70 (*Ccnb1/*APA2), and 50 (*Ccnb1/*APA3); 53 (*Wee2/*APA1) and 132 (*Wee2/*APA2). **c** Different alternative splicing isoforms of *Tcl1* mRNA have different length of poly(A) tails. The gene model and captured isoforms (top) of *Tcl1*. The number of detected isoforms are shown on the right. The poly(A) tail length of detected isoforms is shown in the bottom. The mean length of each isoform poly(A) tail is shown. *p* = 0.03791 between isoform a and c; *p* = 0.014 between isoform b and c. The *p* value was calculated by unpaired and two-sided Student’s *t* test. The number of CCS reads used are 242 (isoform a), 14 (isoform b), 7 (isoform c), and 8 (isoform e).

Alternative polyadenylation (APA) of mRNAs has been shown to play a significant role in many biological processes including mouse oocyte maturation^22^. Our method allows to acquire full-length cDNA sequences with the full information of poly(A) tails. Therefore, it is feasible to analyze the poly(A) tails of different mRNA isoforms from each gene, including APA and alternative splicing. By using the GV oocyte PAIso−seq data, we can map polyadenylation sites of GV oocyte transcripts directly and accurately. When compared with the annotated poly(A) sites in Ensembl mouse genome annotation (mm10, version 92), we identified 3511 genes with two polyadenylation sites (1 APA site), 762 genes with three polyadenylation sites (2 APAs), and 220 genes with more than three polyadenylation sites (≥3 APA sites) (Fig. 3a). These sites overlap well with annotated RNA polyadenylation sites (Fig. 3a). Different isoforms can have different poly(A) tails. For example, we found *Ccnb1* transcripts with three different polyadenylation sites resulting in three different sizes of 3′-UTR. Clearly, the *Ccnb1* transcript APA1 and APA2 isoforms are of similar length of poly(A) tails, while APA3 isoform is of significantly longer poly(A) tails in GV oocytes (Fig. 3b). This is consistent with a previous study of *Ccnb1* poly(A) tail length in a different stage of oocyte development^22^. As another example, we found that the *Wee2*, a key MPF (maturation (M‐phase)-promoting factor) inhibitory kinase necessary for maintaining meiotic arrest^23^, has two different APA isoforms with significantly different length of poly(A) tails (Fig. 3b).

In addition to APA, PAIso−seq also enables the detection of full RNA isoforms together with poly(A) information. Indeed, we see that different alternatively spliced isoforms can have different length of poly(A) tails. For example, *Tcl1* has been annotated with five different isoforms, out of which we detected four in PAIso−seq with different length of poly(A) tails in GV oocytes (Fig. 3b). These results demonstrate that PAIso−seq is a powerful tool that enables the study of isoform-specific poly(A) tails.

### Tail length association with translation in mouse GV oocytes

**Fig. 4:**
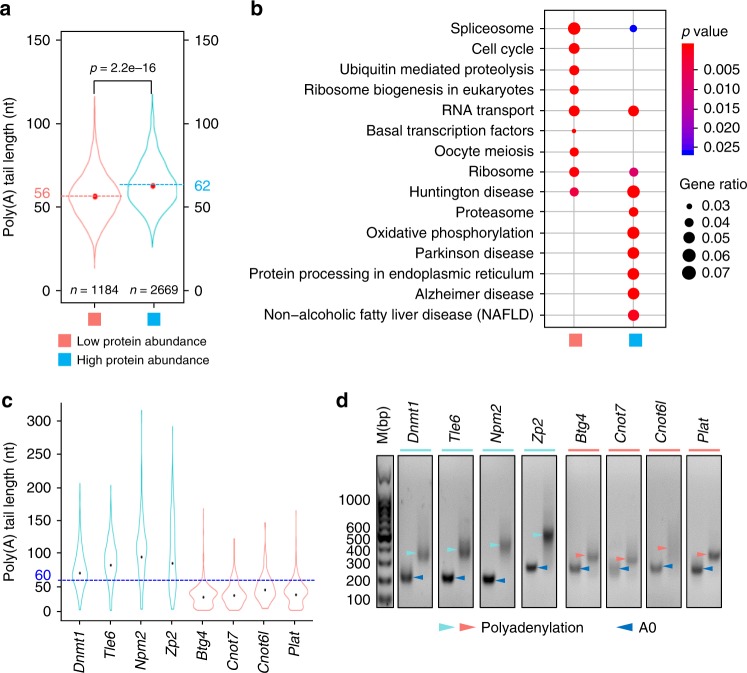
The length of poly(A) tail positively correlates with protein level. **a** Violin plot for poly(A) tail length distribution of low protein abundance and high protein abundance genes (genes with at least ten detected transcripts are included in the analysis). The two dotted lines represent the mean lengths of poly(A) tails of low protein abundance genes (pink) and high protein abundance genes (light blue). The *p* value was calculated by unpaired and two-sided Student’s *t* test. **b** Functional categorization of genes in high protein abundance gene and low protein abundance gene clusters by the KEGG pathway analysis (*p* value cutoff = 0.05). The *p* value is calculated by hypergeometric test. **c** Poly(A) tail length distributions for four high protein abundance genes (*Dnmt1*, *Tle6*, *Npm2*, and *Zp2*, cyan) and four low protein abundance genes (*Btg4*, *Cnot7*, *Cnot6l*, and *Plat*, pink). A blue dotted line indicating 60 nt used to help visualization of the poly(A) tail length difference between high protein abundance and low protein abundance gene groups. The black points indicate the mean poly(A) tail length of each gene. The number of CCS reads used are 390 (*Dnmt1*), 287 (*Tle6*), 146 (*Npm2*), 144 (*Zp2*), 685 (*Btg4*), 94 (*Cnot7*), 73 (*cnot6l*), and 413 (*Plat*). **d** Validation of the poly(A) tail length of the genes shown in Fig. 3c by PAT assay. The dark blue arrowheads represent bands with no poly(A) tail (A0), and the cyan (high protein abundance) and the pink (low protein abundance) arrowheads represent bands with poly(A) tail (polyadenylation). M, marker. Due to additional G tailing and adaptor sequence, the length of polyadenylation PCR products minus A0 products is at least 35 bp longer than the actual poly(A) tails^43^. Source data are provided as a Source Data file.

Many important events happen in the development of oocytes and the very-early-stage embryos, including mRNA and protein storage during oocyte maturation, zygotic genome activation, and maternal mRNA/protein clearance in early embryos. These processes highly depend on mRNAs and proteins stored in the oocyte, which gradually accumulate during oogenesis. Translational control of several mRNAs by poly(A) tail length has been demonstrated in mouse oocytes^5,14,16^. However, it remains unknown wchether it is true transcriptome wide. Wang et al.^24^ previously reported proteome of mouse GV oocytes. Based on the protein profile of GV oocytes, we divided maternal transcripts into two categories: one is of low protein abundance (1184 genes, CCS ≥10, not detected in the mass-spec analysis), the other one is of high protein abundance (2669 genes, CCS ≥10, detected in the mass-spec analysis) (Fig. 4a). By comparing the poly(A) tail length of these two categories, we found that the high protein abundance group has a mean poly(A) tail length of 62 nt, which is significantly longer than that of low protein abundance group at 56 nt (Fig. 4a). The positive correlation between poly(A) tail length and protein level suggests that longer poly(A) tails promote translation in mouse GV oocytes. By KEGG (Kyoto Encyclopedia of Genes and Genomes) pathway analysis, we found that high protein abundance group genes were associated with the ongoing functionality of GV oocytes, such as proteasome and protein processing in endoplasmic reticulum, while low protein abundance group genes were related to will-do functionality of GV oocytes, such as cell cycle and oocyte meiosis (Fig. 4b). Therefore, it suggests that the transcripts of high protein abundance group genes have longer poly(A) tails for efficient translation at the moment, while the transcripts of the low protein abundance group genes have shorter poly(A) tails for storage at the moment. For example, mRNAs of *Dnmt1*, *Tle6*, *Npm2*, and *Zp2*, which have been shown to be actively translated in GV oocytes^25–28^, are with poly(A) tail lengths longer than 60 nt (Fig. 4c). In contrast, *Btg4*, *Cnot7*, *Cnot6l*, and *Plat*, which are well known as dormant maternal mRNAs with lower protein levels in GV oocytes^5,14,29^, are with poly(A) tail lengths shorter than 60 nt (Fig. 4c). These transcripts will be further polyadenylated for efficient translation at later stages of development^5,14,16,30,31^.

To further validate PAIso−seq data, we carried out PAT assay on these eight individual genes as shown in Fig. 3c by using RNA from GV-stage oocytes. The poly(A) tail length determined by PAT assay showed highly similar patterns to those from PAIso−seq (Fig. 4d), confirming that *Dnmt1*, *Tle6*, *Npm2*, and *Zp2* truly have longer poly(A) tails than *Btg4*, *Cnot7*, *Cnot6l*, and *Plat*, demonstrating that the PAIso−seq can measure poly(A) tail lengths accurately, providing the global association between protein synthesis and the mRNA poly(A) tail length in mouse GV oocytes.

### Widespread non-adenosine residues within RNA poly(A) tails

**Fig. 5:**
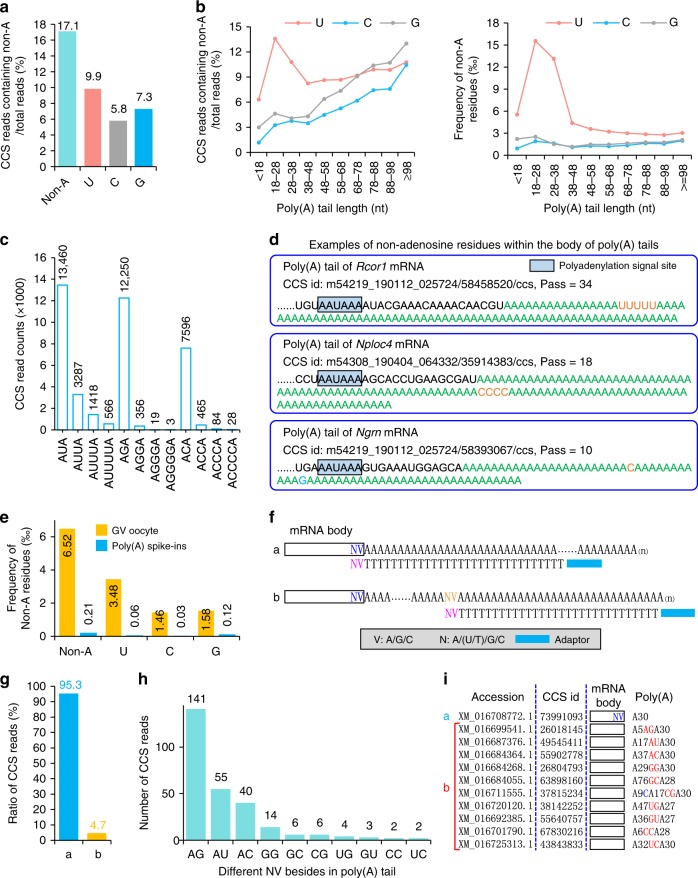
Widespread non-adenosine residues within the body of poly(A) tails. **a** Frequency of CCS reads containing internal non-A nucleotides within poly(A) tail. **b** The ratio of CCS reads containing internal non-A nucleotides (left panel) and frequency of non-adenosine residues in CCS reads of different poly(A) tail length (right panel). **c** Mono- and oligo-non-adenosine residue (U, C, and G) counts. **d** Three examples of CCS reads with non-adenosine residues in the body of poly(A) tails, *Rcor1* (Pass = 34), *Nploc4* (Pass = 18), and *Ngrn* (Pass = 10). **e** The frequency of non-A residues in GV oocyte and poly(A) spike-in data. **f** Hypothetic models of CCS reads with T30VN RT primer anchored at the end of 3′-UTR (**a**) or within the body of poly(A) tails (**b**). **g** Percentage of two different T30VN RT primer-anchoring models as revealed by CCS reads from Iso-seq data. **h** The frequency of different NV-anchoring sites detected within the body of poly(A) tails. The number of the detected events are shown above each bar. **i** Examples of CCS reads with T30VN RT primer anchored at the end of 3′-UTR (**a**) or within the body of poly(A) tails (**b**). The accession number of the CCS reads is shown on the left. The CCS read i.d. is shown in the middle. The CCS read model is shown on the right. The number after A means oligo A with the given number of adenosines.

RNA poly(A) tails were thought to be only composited of A residues. Through TAIL-seq, pervasive 3′ end G and U modifications have been found in RNA poly(A) tails with a vital role in mRNA stability in human cell lines^2,3^. By using the same method, 3′ end of maternal transcripts with short poly(A) tail length have been shown to be uridylated in mouse GV oocytes^10^. However, the non-T signal cannot be accurately called within a long stretch of T by using the base-calling algorithm in TAIL-seq method. Therefore, non-T signal can only be called at the very 3′ end by using TAIL-seq. Our method does not have this limitation. Moreover, multiple passes of a single template generate highly accurate CCS of a transcript, including the bases within poly(A) tails. Therefore, PAIso−seq gives us the opportunity to analyze the detailed base composition within poly(A) tails. We used a high threshold requiring at least 10 passes for a single molecule to ensure the accuracy of the sequence called^19,32^. Surprisingly, there are widespread U, G, and C inside the body of mRNA poly(A) tails besides that which can be seen in the 3′ end within 17% of the transcripts (Fig. 5a). When we looked into the Us, Gs, or Cs in poly(A) tails of different length, we saw that U modifications are more frequent in transcripts with short tails, while G and C modifications are more frequent in relatively long tails (Fig. 5b). In general, the modifications are more frequently seen near the 5′ end of poly(A) tails, whereas G modification has another obvious enriched position near the 3′ end of the tails (Supplementary Fig. 4). Besides, we found that there are two, three, and even four (although with relatively low frequency) continuous non-adenosine residues inside the poly(A) tails, in addition to the single U, C, or G that are more frequent (Fig. 5c). As examples, we detected the poly(A) tails of *Rcor1* (Pass = 34), *Nploc4* (Pass = 18), and *Ngrn* (Pass = 10) transcripts with multiple non-adenosine residues within the body of poly(A) tails (Fig. 5c). The ratio of transcripts containing non-A modifications of genes shows good correlation between replicates (Supplementary Fig. 5).

To make sure that the non-A modification seen here is not caused by errors introduced during library construction, we checked the data of synthetic poly(A) + cDNA spike-in after RT during library preparation step. The result showed about 0.02% mismatches within spike-in poly(A) sequences that have passes ≥10 (59 out of 283,007 bases) (Fig. 5e). This is much lower than about 0.7% non-A modifications seen in poly(A) tails. Therefore, the non-A modifications we observe cannot be from steps after RT. The RT enzymes used in this method, SuperScript II, are known to introduce about 1/42,000 (0.0024%) mismatches during RT reaction^33,34^. Although RT enzymes might have increased the chance to introduce frameshift when dealing with homopolymers^33^, it will not generate more mismatches. Therefore, there are poly(A) tail internal modifications missed in previous analysis due to technical limitations, although a very small portion of them detected here might be caused by RT mistakes. To further confirm that the non-A residues are not artifacts, we looked into coding sequences with oligo A and oligo T tracks more than ten bases. These homopolymeric sequences are coded in the genome; therefore, if there are mismatches in the data they are likely caused by errors introduced during RT step or sequencing step. There are eight such regions within the genome covered by 20 reads in our dataset. There are in total 243 bases sequenced, no SNV (single-nucleotide variation) is detected, indicating few if no errors are introduced during RT steps.

To validate the existence of non-A modifications by using sequencing-independent information, we looked into the base-pairing information between RNA and RT primers. Traditionally, full-length cDNA Iso-seq on PacBio platform used 5′-adaptor-T30VN-3′ oligo as RT primer for reverse transcription; V (A, C, or G) and N (A, T, C, or G) are used to anchor the RT primer to the end of 3′-UTR to discard poly(A) tails during reverse transcription. We reason that the RT primer can also anchor to the non-A residues in the middle of poly(A) tails if non-A residues are present in the middle of the tail. This implies a testable hypothesis that base pairing between poly(A) internal non-A residue and VN in RT primer will result in inclusion of poly(A) sequences before VN (Fig. 5f). To test this, we randomly chose a recently published Iso-seq dataset from pepper (experiment CRX041331 under accession number CRA001412)^35^. Indeed, the result is as what we hypothesized. There are about 5% transcripts showing VN base pairs with nucleotides within the middle of the poly(A) tails (Fig. 5g), where single non-A is more frequent than double non-A (Fig. 5h). Examples of these middle RT primer-anchored reads are shown in Fig. 5i. The RT primer base pairing takes place before RT reaction, supporting that there are non-A residues within the body of poly(A) tails. Although RT is very inefficient (2–6 × 10^−4^ relative normal rate) to extend on primers with mismatches on the very 3′ end^36^, we cannot exclude the possibility that some of the middle RT primer anchoring events detected here resulted from mispriming at the middle of pure A tails. These data validate that the non-A modifications within poly(A) tails is not likely caused by sequencing or library preparation artifacts.

These findings indicate that PAIso−seq allows accurate decomposition of poly(A) tails, revealing widespread U, G, and C modifications within the body of mRNA poly(A) tails, indicating that mRNA poly(A) tails are far more complex than what was previously thought.

### Poly(A) tails in other cells

The above poly(A) tail length and base composition is about one single-cell type, the mouse GV oocyte. To validate the PAIso−seq method in other cell types, we employed the rat liver sample that have never been analyzed at the mRNA poly(A) tail aspect and obtained PAIso−seq data successfully (Supplementary Fig. 1a, c). We can see that the rat liver sample has a very different pattern of global transcript poly(A) tail length compared with mouse GV oocytes (Supplementary Fig. 6a and Fig. 2a). The rat liver sample also contains a substantial part of transcripts with non-A modifications within poly(A) tails (Supplementary Fig. 6b). These data confirm that PAIso−seq is widely applicable to samples from different cells and different species and will be a powerful tool to dissect the regulation through RNA poly(A) tails in diverse biological processes.

### Single-cell PAIso−seq

**Fig. 6:**
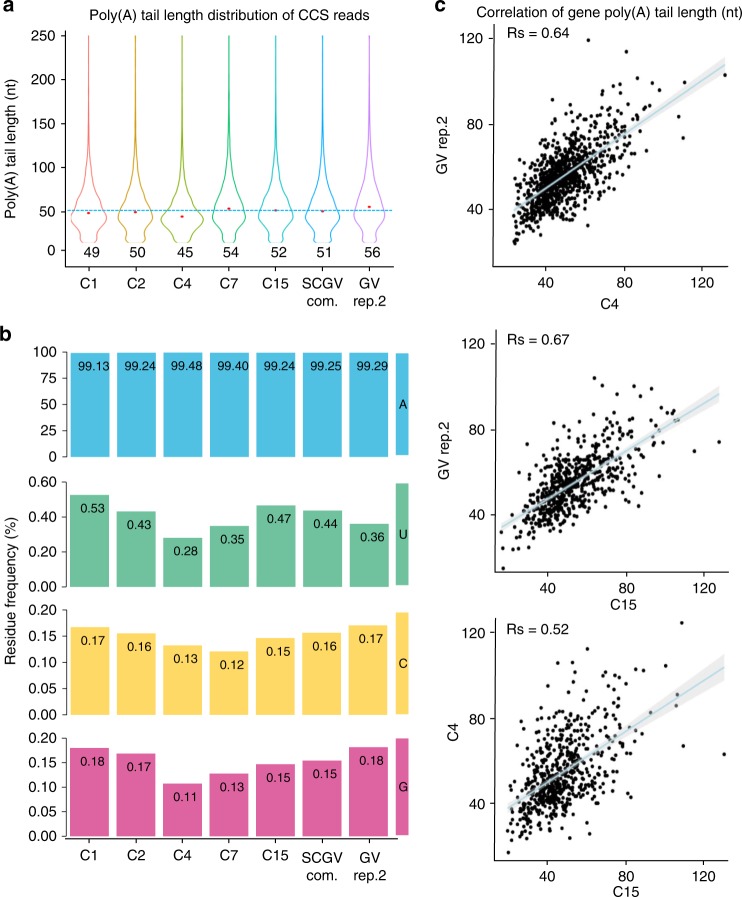
Single-cell PAIso−seq for GV oocytes. **a** Global poly(A) tail length distributions of all detected CCS reads (Pass ≥10) in single GV oocyte C1 (cell 1), C2, C4, C7, C15, and GV rep.2, and single GV oocyte-combined (SCGV com.) datasets. The median length of each poly(A) tail of a transcript is shown as the red dot and the number below the violin plot. **b** The frequency of non-A residues in single GV oocytes C1, C2, C4, C7, C15, GV rep.2, and SCGV com. datasets. **c** The Spearman’s correlation of gene poly(A) tail length between single GV oocyte C4 and GV rep.2 (top panel, *n* = 928), single GV oocyte C15 and GV rep.2 (middle panel, *n* = 566), single GV oocyte C15 and C4 datasets (bottom panel, *n* = 597) datasets. The blue line represents linear regression line. The light-gray area represents confidence interval of the regression. Genes with at least four transcripts in each of the datasets are included in the analysis.

The PAIso−seq library construction steps are highly efficient. We think that it might be applicable to single cells. Therefore, we tested PAIso−seq in 15 single GV oocyte samples with barcoded end-extension primer. The samples are pooled for sequencing. The single-cell data showed similar transcript abundance and poly(A) length distribution pattern as bulk sample (Fig. 2a–c). The 15 single-cell data are also comparable to bulk cell data in measuring both poly(A) tail length and non-A modifications (Fig. 6a, b). At individual gene level, we can see that poly(A) tail length measured from each single cell correlates well with that from bulk cells (Fig. 6c and Supplementary Fig. 7). These data demonstrate that PAIso−seq is capable to deal with a single GV oocyte containing about 0.3–0.5 ng of total RNA^37^, offering an opportunity to study global RNA poly(A) tails in rare cells.

## Discussion

RNA poly(A) tails, common and vital modifications for mRNA and lncRNAs, were discovered in 1971^38–40^. However, despite rich knowledge in poly(A) biogenesis and regulation, the detailed composition of genome-wide poly(A) tails has remained elusive due to technical limitations. Our PAIso−seq is based on PacBio single-molecule sequencing that is capable of analyzing homopolymers. PacBio circular single-molecule sequencing makes a single molecule to be read multiple passes, which enables highly accurate consensus sequence generation. We require a pass number of 10 or more to ensure the accuracy of base calling. Therefore, our PAIso−seq technology provides an accurate method in analyzing the length and base composition of poly(A) tails. As PacBio allows read length up to 45 kb, this enables us to measure poly(A) tails with very long length. We can reliably detect transcripts with long poly(A) tails among different replicates, which are larger than 231 nt in length that TAIL-seq can handle and previously thought of as a 250-nt upper limit^8^ (Fig. 2f), although the biological significance awaits further investigation. There are also rare cases of poly(A) tails as long as 13 kb in length. The 13-kb tail is not detected in other replicates, indicating very low abundance. As the long reads contain full cDNA sequence of the transcript, it enables us to look into the isoform-specific poly(A) tails, including alternative splicing and APA (Fig. 3). This will give insights into the coordinated regulation of multiple steps of mRNA maturation.

More interestingly, we revealed that there are widespread non-adenosine residues within the body of mRNA poly(A) tails in mammals (Fig. 5). Chemically modified bases within the poly(A) tails might be another possible source to introduce U, C, or G substitutions during the RT step, although this is very unlikely. A recent report by using direct poly(A)-seq on Illumina platform also reveals that G residue in transcripts of more than 15% detected transcripts with good poly(A) tail sequencing quality, supporting the existence of non-A modifications within the body of mRNA poly(A) tails in multiple species^41^. Non-A modifications have been previously identified at the very 3′ end of mRNA poly(A) tails^2,3,8^. The U and G residues at the very end of poly(A) tails have been shown to be important for the regulation of mRNA stability that either promote or inhibit the degradation of the poly(A) tails^2,3^. It will be very interesting to investigate the global dynamics of poly(A) tails during development, as well as the regulatory functions of the non-A residues in the body of RNA poly(A) tails in the future. This will not be limited to mouse reproductive system, as we can clearly see distinct global poly(A) tail length and poly(A) tail body non-A modification pattern from other tissue and other species (Supplementary Fig. 6).

A similar method called full-length poly(A) and mRNA sequencing (FLAM-seq) was published when this work was under review. This method also uses PacBio sequencing to deal with the homopolymer problem^42^. Indeed, FLAM-seq and PAIso−seq agree well with each other in the accuracy of tail length quantification and non-A modification detection, demonstrating the power of the two methods. In addition to synthetic cDNA spike-ins used here, Legnini et al.^42^ also used synthetic RNA to verify the accuracy of the method and reached the same conclusion as what we found here by using synthetic cDNAs, confirming that RT step had minimal impact to the accuracy of poly(A) tail analysis. The main difference between these two methods is the way to add adaptor sequence to the very end of poly(A) tails. PAIso−seq uses templated extension, while FLAM-seq uses G/I tailing. Based on the tests at this moment, PAIso−seq may be more sensitive that it can handle single GV oocytes, while FLAM-seq needs 500 ng of total RNA.

During application of PAIso−seq, there are two limitations we know currently. First, PAIso−seq cannot accurately quantify the frequency of non-A modifications at the very last 3′-end base, because 3′ end G, C, and U can pair to the 3′ base of the template adaptor sequence and will be lost during data analysis. This will also cause a minimal number of additional A introduced during pairing of the extension primer. However, this bias shall be much lower than poly(A) + RNA purification by using poly(T) resin. The other limitation is the cost to achieve high coverage, because current PacBio sequencing cost (about 5 dollars per 1-K reads on Sequel instrument used in this study) is 1000 times higher per read than that of Illumina sequencing (about 5 dollars per 1-M 150-bp pair-end reads). The correlation between different replicates as we shown in Fig. 2c can be expected to be further improved if we sequence more reads. PacBio sequencing cost has dramatically decreased recently. Recently shipped Sequel II instrument has already cut the per-read cost by 70% (about 1.5 dollars per 1-K reads on Sequel II).

TAIL-seq, PAL-seq, and FLAM-seq methods need microgram level of total RNA to start with, which is not applicable to many primary or patient samples^1,8,42^. The PAIso−seq method we presented in this study used end extension coupled with template switching during reverse transcription, which is highly efficient. The good performance of the method in single GV oocyte analysis enables the application of this method to precious rare samples.

In summary, PAIso−seq is accurate and sensitive in analyzing poly(A) tails genome wide. By using this method, we can analyze isoform-specific poly(A) tail regulation. Moreover, we revealed widespread non-adenosine modifications within the body of poly(A) tails, which are masked by previous methods in analyzing poly(A) tails. The single-oocyte sensitivity of this method paves the way to analyze rare primary and patient samples. These findings along with the PAIso−seq method may open the door to understand the function and regulation of poly(A) tails.

## Methods

### Animals and collection of oocytes

Mice were purchased from Beijing Vital River Laboratory Animal Technology Co., Ltd., and maintained in compliance with the guidelines of the Animal Care and Use Committee of the Institute of Genetics and Development Biology, Chinese Academy of Sciences (CAS). For preparation of GV oocytes, 7–8-week-old CD1 (ICR) mice were intraperitoneally injected with 10 U of pregnant mare serum gonadotropin (Prospec). Ovaries were punctured with a 30-gauge needle to release GV oocytes. Oocytes were washed with M2 medium (Sigma) and finally with phosphate-buffered saline (PBS) containing 0.1% bovine serum albumin (PBS with BSA) three times.

### RNA isolation

Total RNA was extracted with Direct-zol RNA MicroPrep (Zymo Research) according to the manufacturer’s instructions. Briefly, oocytes were lysed directly in 500 μl of TRIzol reagent (Ambion) and mixed thoroughly; then 500 μl of 100% ethanol was added and mixed thoroughly. The mixture was transferred into a Zymo-Spin IC Column and centrifuged. After washing, the RNA was eluted by adding 10–30 μl of nuclease-free water directly to the column matrix. Prepared RNA was stored at −80 °C or used immediately.

### PAIso−seq library construction

Total RNA (about 100 ng, RNA integrity number values of 9.1 and 8.5, respectively) from GV-stage oocytes is used for end extension with dNTP and Klenow fragment (exo^−^, NEB) on a dU-containing DNA template (BC3-2dU primer) (Supplementary Table 1) at 37 °C for 1 h. dUs were digested by USER enzymes (NEB) at 37 °C for 30 min. End-extended RNAs (>200 nt) was cleaned up and concentrated with RNA Clean & Concentrator-5 Kit (Zymo Research): the sample was mixed thoroughly with 50 μl of RNA-binding buffer and 50 μl of 100% ethanol; the mixture was transferred into a Zymo-Spin IC Column and centrifuged; after washing, the nucleic acids were eluted by adding 6–8 μl of nuclease-free water directly to the column matrix and centrifuging. Prepared RNA was stored at −80 °C or used immediately. The cleaned end-extended RNA was reverse transcribed with template switching similarly to Smart-seq2 method with RT primer and TSO^9^ (Supplementary Table 1). PCR amplification was performed by using KAPA HiFi HotStart ReadyMix (KAPA Biosystems) with PCR primers (Supplementary Table 1). Amplified cDNA products were size selected by Pure PB beads (1× beads for cDNA more than 200 bp and 0.4× beads for cDNA more than 2 kb cDNA, the two parts of the sample were combined at equal molar ratios for further library construction), made into SMRTbell Template libraries (SMRTbell Template Prep Kit). The libraries were annealed with the sequencing primer and bound to polymerase, and finally the polymerase-bound template was bound to Magbeads and sequenced by using PacBio Sequel instruments at Annoroad or Novogene.

### PAIso−seq library construction for single GV oocyte

Single GV oocyte was washed by using PBS with 1% BSA three times. Single GV oocyte was lysed in buffer containing 0.5 μl of RNase inhibitor to 10.5 μl of 0.2% (vol/vol) Triton X-100 at 80 °C for 5 min. The mixture was then used for end extension with dNTP and Klenow fragment (exo^−^, NEB) on a dU-containing DNA template (Supplementary Table 1) at 37 °C for 1 h. Fifteen single GV oocytes were barcoded with different dU-containing DNA templates (Supplementary Table 1). dUs were digested by USER enzymes (NEB) at 37 °C for 30 min. End-extended RNAs (>200 nt) was cleaned and concentrated with RNA Clean & Concentrator-5 Kit (Zymo Research). Fifteen cleaned end-extended RNAs were combined and proceeded with regular PAIso−seq library construction.

### Poly(A) spike-ins

The oligos (Supplementary Table 3) used to prepare poly(A) spike-ins were synthesized by Takara Biomedical Technology Co., Ltd. The poly(A) spike-ins were PCR amplified with PSI-*-F and PSI-*-R primers (Supplementary Table 3) with 20 ng of mCherry plasmid template by using KAPA HiFi HotStart ReadyMix (KAPA Biosystems). The PCR reaction was performed in a thermocycler by using a touchdown program: 98 °C for 5 min, 18× (98 °C for 20 s, 68 °C (reduced by 1° each cycle) for 15 s, and 72 °C for 70 s), and then cycled (98 °C for 20 s, 61 °C for 15 s, and 72 °C for 70 s) for appropriate cycles, 72 °C for 10 min. PCR products were gel purified. A plasmid containing mCherry sequence was used as the template for PCR. To distinguish spike-ins with different poly(A) tail length, they were barcoded before the start codon. The complete sequences of the poly(A) spike-ins are included in Supplementary Fig. 2a. Equal amount of the standards with different poly(A) tails were used as spike-ins for sequencing. The recovery rates in the sequencing were similar between standards with different length of poly(A) tails (Supplementary Fig. 2b).

### PAT assay

PAT assay was performed by using a poly(G) tailing method as previously described with minor modifications^43^. Briefly, total RNA was isolated from 500 GV oocytes. Total RNA was incubated with 75 U of yeast poly(A) polymerase (Thermo) in the presence of 0.375 mM guanosine-5′-triphosphate and 0.125 mM inosine triphosphate (at 37 °C for 60 min. cDNA was synthesized by using SuperScript II Reverse Transcriptase (Invitrogen) and PAT-C10T2 primer at 42 °C for 1 h. PAT-PCR was performed using a gene-specific forward primer (Supplementary Table 2) and PAT-PCR-R prime or gene-specific reverse (A0) primer with KAPA HiFi HotStart ReadyMix (KAPA Biosystems). Due to additional G tailing and adaptor sequence, the length of polyadenylation PCR products minus A0 products is at least 35 bp longer than the actual poly(A) tails. PCR products were resolved in a 2% agarose gel. The size of PCR products was also analyzed with Fragment Analyzer (Advanced Analytical).

### Sequencing data processing

CCS reads were generated from subread by using ccs (version 3.4.1), which took multiple subreads of the same SMRTbell molecule and combined them by using a statistical model to produce one highly accurate consensus sequence with the following parameters: --no_polish TRUE, --min_passes 1, --min_predicted_accuracy 0.9, and others default. To extract and clean CCS reads, we matched 22-nt sequence (including the 16-nt sample barcode sequence and the first six bases of the TSO sequence) in CCS reads and the reverse complementary of CCS reads allowing a maximum of two mismatches or indels. The 3′ adapters were trimmed and CCS reads were oriented from 5′ to 3′ with poly(A) tail in the 3′ end. Concatemer reads were detected when primers were found in the middle of the reads, the primer and the sequences ahead of the primer were trimmed. The 5′ cDNA primers were removed when presented in the CCS reads. For library containing multiple samples, CCS reads were extracted separately, and ambiguous reads that could be classified into two or more samples, were discarded. Clean CCS reads were used for downstream analysis. To ensure the accuracy of base calling and tail length calling, all reads used in the downstream analysis are with passes of at least 10 if not specified.

### Poly(A) tail length measurement

Clean CCS reads were aligned to mouse reference genome (mm10) by using minimap2 (v2.15-r905) with the following parameters: -t 20 -ax splice -uf -C5 --cs = short --secondary = no. Alignments with supplementary alignment tag were ignored. The terminal clipped sequence of the CCS reads in the alignment bam file is considered as poly(A) tail when they fulfill all the following criteria: (1) the length of the clipped sequence was no <15 nt; (2) the clipped sequence contained at least five continuous adenosines; (3) the percentage of the non-A residues was <50% and the number of non-A residues were <20. The clipped sequences were considered as poly(A) tails. The poly(A) tail length of the transcript was calculated as the length of the clipped sequence. The poly(A) tail length of a gene was presented by the geometric mean of the poly(A) tail length of transcripts from the same gene, because poly(A) tail length distribution of a gene is a lognormal-like distribution.

### Detection of non-A residues in poly(A) tails

Clean CCS reads with at least ten passes were used for calling non-A residues in poly(A) tails. G, C, and U (presented as T in CCSs) were counted in poly(A) tail of each transcript. The G, C, and U ratio of a transcript were the counts of G, C, and U divided by the poly(A) tail length of this transcript, respectively. The non-adenosine ratio of a transcript is the sum of G, C, and U ratio. The method of calculating the non-A ratio of a gene was the same as transcripts, except to combine all transcripts from the same gene.

### Analysis of synthetic poly(A) spike-ins

Pooled synthetic cDNAs with defined poly(A) tail lengths of 10, 30, 50, 70, and 100 nt were separated according to their barcode sequence. CCS reads were oriented from 5′ to 3′ such that each reads starts with the 5′ primer, followed by the CDS (coding sequence) of mCherry, the poly(A) tail, and the 3′ primer. Full-length reads containing all the above features were included in the analysis. The sequences between mCherry and 3′ primer were considered as poly(A) tail used to calculate the lengths and non-adenosine ratios of different spike-ins.

### Assigning CCS reads to genes

Clean CCS reads were aligned to mouse reference genome (mm10) by using minimap2 (v2.15-r905) with parameters -t 20 -ax splice -uf -C5 --cs = short --secondary = no. Mouse exons were extracted from the Ensembl genome annotation file, overlapped exons were merged, and duplicated exons were removed. CCSs were assigned to genes according to the mapping between CCSs and exons. CCSs assigned to multiple genes were discarded.

### Detection of polyadenylation sites

Polyadenylation analysis was performed by using the TAPIS pipeline^44^. Clean CCS reads with poly(A) tail trimmed were aligned to mouse reference genome (mm10) by using GMAP; APA events were then analyzed by using run_tapis.py script.

### KEGG enrichment analysis

To compare the enriched functional categories between low protein abundance genes and high protein abundance genes, KEGG enrichment analysis was performed by using the compareCluster function in the R/Bioconductor package clusterProfiler based on a hypergeometric distribution by using a background list of all proteins in the *Mus musculus* annotation database.

### Genome and annotation

The genome sequence and annotations used in this study are from the following links. Mouse genome file (ftp://ftp.ensembl.org/pub/release-92/fasta/mus_musculus/dna/Mus_musculus.GRCm38.dna_rm.primary_assembly.fa.gz), mouse genomic annotation file (ftp://ftp.ensembl.org/pub/release-92/gtf/mus_musculus/Mus_musculus.GRCm38.92.gtf.gz), and Poly(A) sites annotation (https://polyasite.unibas.ch/download/clusters/GRCm38-96/2-0/clusters.bed.gz).

### Reporting summary

Further information on research design is available in the Nature Research Reporting Summary linked to this article.

## Supplementary information

Supplementary Information

Reporting Summary

## Data Availability

The PAIso−seq CCS reads have been deposited into NCBI Sequence Read Archive under the accession number PRJNA529588. The SRA accession numbers of GV rep.1 and GV rep.2 are SRR8798075 and SRR9130368, respectively. The SRA accessions of 15 single GV oocyte PAIso−seq data are SRR9130400, SRR9130401, SRR9130402, SRR9130403, SRR9130404, SRR9130405, SRR9130406, SRR9130407, SRR9130408, SRR9130409, SRR9130410, SRR9130411, SRR9130412, SRR9130413, and SRR9130414. All other data are available upon request. The source data underlying Figs. 1b and 4d are provided as a Source Data file. Any other relevant data are available from the authors upon request.

## Source data

Source Data

## Code availability

Key scripts used to process and analyze PAIso−seq data are available on GitHub (https://github.com/niehu2018/GV_oocyte_PAIsoSeqAnalysis).
