# Supplementary material for: Poly(A) inclusive RNA isoform sequencing (PAIso−seq) reveals wide-spread non-adenosine residues within RNA poly(A) tails
Source: Nat Commun. 2019 Nov 22;10:5292. doi: 10.1038/s41467-019-13228-9 (PMC6876564; doi:10.1038/s41467-019-13228-9)
Supplement: Supplementary file 1 — Supplementary Information [file 41467_2019_13228_MOESM1_ESM.pdf]

Poly(A) inclusive RNA isoform sequencing (PAIso-seq) reveals wide-spread non-adenosine residues within RNA poly(A) tails

Liu, *el al.*

Supplementary Information

The supplementary information consists of 7 supplementary figures and 4 supplementary tables.

SUPPLEMENTARY FIGURES

a

Samples	CCS read counts
GV rep.1	79,994
GV rep.2	227,902
SCGV com.	191,023
Rat liver	83,237

b

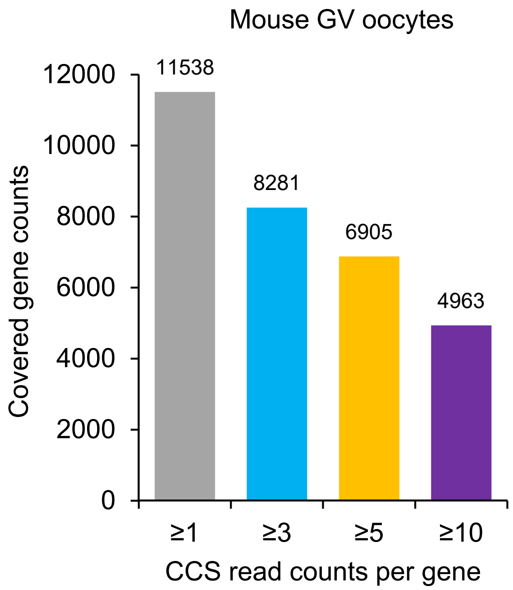

c

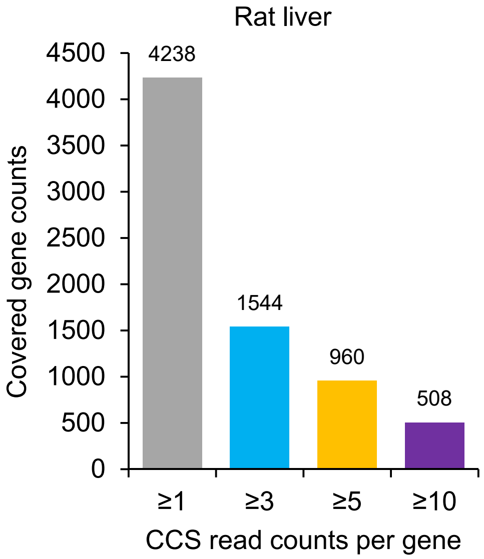

Supplementary Figure 1. Transcripts captured by PAIso-seq in mouse GV oocytes and Rat liver.

(a) CCS read counts in three replicates of GV oocytes and Rat liver samples. One transcript here refers to one CCS read that can map to the corresponding transcriptome or genome.

(b-c) Number of genes at given coverage in mouse GV oocytes (B), Rat liver (C).

a

TSO-barcode-mcherry-polyA-TSO

AAGCAGTGGTATCAACGCAGAGTACCGCTGCGAGAGACAGTATGGTGAGCAAGGGCGAGGAGGATAACATGGCCATCATCAAGGAGTTCATGCGCTTCAAGGTGCACATGGAGGGCTCCGTGAACGGCCACGAGTT
CGAGATCGAGGGCGAGGGCGAGGGCCGCCCCTACGAGGGCACCCAGACCGCCAAGCTGAAGGTGACCAAGGGTGGCCCCCTGCCCTTCGCCTGGGACATCCTGTCCCCTCAGTTCATGTACGGCTCCAAGGCCTAC
GTGAAGCACCCCGCCGACATCCCCGACTACTTGAAGCTGTCCTTCCCCGAGGGCTTCAAGTGGGAGCGCGTGATGAACTTCGAGGACGGCGGCGTGGTGACCGTGACCCAGGACTCCTCCCTGCAGGACGGCGAGT
TCATCTACAAGGTGAAGCTGCGCGGCACCAACTTCCCCTCCGACGGCCCCGTAATGCAGAAGAAGACCATGGGCTGGGAGGCCTCCTCCGAGCGGATGTACCCCGAGGACGGCGCCCTGAAGGGCGAGATCAAGCA
GAGGCTGAAGCTGAAGGACGGCGGCCACTACGACGCTGAGGTCAAGACCACCTACAAGGCCAAGAAGCCCGTGCAGCTGCCCGGCGCCTACAACGTCAACATCAAGTTGGACATCACCTCCCACAACGAGGACTAC
ACCATCGTGGAACAGTACGAACGCGCCGAGGGCCGCCACTCCACCGGCGGCATGGACGAGCTGTACAAGTGAGTACTCTGCGTTGATACCACTGCTT (0A)

AAGCAGTGGTATCAACGCAGAGTACGCTCGTCGCGCGCACAATGGTGAGCAAGGGCGAGGAGGATAACATGGCCATCATCAAGGAGTTCATGCGCTTCAAGGTGCACATGGAGGGCTCCGTGAACGGCCACGAGTT
CGAGATCGAGGGCGAGGGCGAGGGCCGCCCCTACGAGGGCACCCAGACCGCCAAGCTGAAGGTGACCAAGGGTGGCCCCCTGCCCTTCGCCTGGGACATCCTGTCCCCTCAGTTCATGTACGGCTCCAAGGCCTAC
GTGAAGCACCCCGCCGACATCCCCGACTACTTGAAGCTGTCCTTCCCCGAGGGCTTCAAGTGGGAGCGCGTGATGAACTTCGAGGACGGCGGCGTGGTGACCGTGACCCAGGACTCCTCCCTGCAGGACGGCGAGT
TCATCTACAAGGTGAAGCTGCGCGGCACCAACTTCCCCTCCGACGGCCCCGTAATGCAGAAGAAGACCATGGGCTGGGAGGCCTCCTCCGAGCGGATGTACCCCGAGGACGGCGCCCTGAAGGGCGAGATCAAGCA
GAGGCTGAAGCTGAAGGACGGCGGCCACTACGACGCTGAGGTCAAGACCACCTACAAGGCCAAGAAGCCCGTGCAGCTGCCCGGCGCCTACAACGTCAACATCAAGTTGGACATCACCTCCCACAACGAGGACTAC
ACCATCGTGGAACAGTACGAACGCGCCGAGGGCCGCCACTCCACCGGCGGCATGGACGAGCTGTACAAGTGAAAAAAAAAAAGTACTCTGCGTTGATACCACTGCTT (10A)

AAGCAGTGGTATCAACGCAGAGTACACAGTGCGCTGTCTATATGGTGAGCAAGGGCGAGGAGGATAACATGGCCATCATCAAGGAGTTCATGCGCTTCAAGGTGCACATGGAGGGCTCCGTGAACGGCCACGAGTT
CGAGATCGAGGGCGAGGGCGAGGGCCGCCCCTACGAGGGCACCCAGACCGCCAAGCTGAAGGTGACCAAGGGTGGCCCCCTGCCCTTCGCCTGGGACATCCTGTCCCCTCAGTTCATGTACGGCTCCAAGGCCTAC
GTGAAGCACCCCGCCGACATCCCCGACTACTTGAAGCTGTCCTTCCCCGAGGGCTTCAAGTGGGAGCGCGTGATGAACTTCGAGGACGGCGGCGTGGTGACCGTGACCCAGGACTCCTCCCTGCAGGACGGCGAGT
TCATCTACAAGGTGAAGCTGCGCGGCACCAACTTCCCCTCCGACGGCCCCGTAATGCAGAAGAAGACCATGGGCTGGGAGGCCTCCTCCGAGCGGATGTACCCCGAGGACGGCGCCCTGAAGGGCGAGATCAAGCA
GAGGCTGAAGCTGAAGGACGGCGGCCACTACGACGCTGAGGTCAAGACCACCTACAAGGCCAAGAAGCCCGTGCAGCTGCCCGGCGCCTACAACGTCAACATCAAGTTGGACATCACCTCCCACAACGAGGACTAC
ACCATCGTGGAACAGTACGAACGCGCCGAGGGCCGCCACTCCACCGGCGGCATGGACGAGCTGTACAAGTGAAAAAAAAAAAAAAAAAAAAAAAAAAAAAAAGTACTCTGCGTTGATACCACTGCTT (30A)

AAGCAGTGGTATCAACGCAGAGTACTCACACTCTAGAGCGAATGGTGAGCAAGGGCGAGGAGGATAACATGGCCATCATCAAGGAGTTCATGCGCTTCAAGGTGCACATGGAGGGCTCCGTGAACGGCCACGAGTT
CGAGATCGAGGGCGAGGGCGAGGGCCGCCCCTACGAGGGCACCCAGACCGCCAAGCTGAAGGTGACCAAGGGTGGCCCCCTGCCCTTCGCCTGGGACATCCTGTCCCCTCAGTTCATGTACGGCTCCAAGGCCTAC
GTGAAGCACCCCGCCGACATCCCCGACTACTTGAAGCTGTCCTTCCCCGAGGGCTTCAAGTGGGAGCGCGTGATGAACTTCGAGGACGGCGGCGTGGTGACCGTGACCCAGGACTCCTCCCTGCAGGACGGCGAGT
TCATCTACAAGGTGAAGCTGCGCGGCACCAACTTCCCCTCCGACGGCCCCGTAATGCAGAAGAAGACCATGGGCTGGGAGGCCTCCTCCGAGCGGATGTACCCCGAGGACGGCGCCCTGAAGGGCGAGATCAAGCA
GAGGCTGAAGCTGAAGGACGGCGGCCACTACGACGCTGAGGTCAAGACCACCTACAAGGCCAAGAAGCCCGTGCAGCTGCCCGGCGCCTACAACGTCAACATCAAGTTGGACATCACCTCCCACAACGAGGACTAC
ACCATCGTGGAACAGTACGAACGCGCCGAGGGCCGCCACTCCACCGGCGGCATGGACGAGCTGTACAAGTGAAAAAAAAAAAAAAAAAAAAAAAAAAAAAAAAAAAAAAAAAAAAAAAAAAAAGTACTCTGCGTTGA
TACCACTGCTT (50A)

AAGCAGTGGTATCAACGCAGAGTACTCACATATGTATACATATGGTGAGCAAGGGCGAGGAGGATAACATGGCCATCATCAAGGAGTTCATGCGCTTCAAGGTGCACATGGAGGGCTCCGTGAACGGCCACGAGTT
CGAGATCGAGGGCGAGGGCGAGGGCCGCCCCTACGAGGGCACCCAGACCGCCAAGCTGAAGGTGACCAAGGGTGGCCCCCTGCCCTTCGCCTGGGACATCCTGTCCCCTCAGTTCATGTACGGCTCCAAGGCCTAC
GTGAAGCACCCCGCCGACATCCCCGACTACTTGAAGCTGTCCTTCCCCGAGGGCTTCAAGTGGGAGCGCGTGATGAACTTCGAGGACGGCGGCGTGGTGACCGTGACCCAGGACTCCTCCCTGCAGGACGGCGAGT
TCATCTACAAGGTGAAGCTGCGCGGCACCAACTTCCCCTCCGACGGCCCCGTAATGCAGAAGAAGACCATGGGCTGGGAGGCCTCCTCCGAGCGGATGTACCCCGAGGACGGCGCCCTGAAGGGCGAGATCAAGCA
GAGGCTGAAGCTGAAGGACGGCGGCCACTACGACGCTGAGGTCAAGACCACCTACAAGGCCAAGAAGCCCGTGCAGCTGCCCGGCGCCTACAACGTCAACATCAAGTTGGACATCACCTCCCACAACGAGGACTAC
ACCATCGTGGAACAGTACGAACGCGCCGAGGGCCGCCACTCCACCGGCGGCATGGACGAGCTGTACAAGTGAAAAAAAAAAAAAAAAAAAAAAAAAAAAAAAAAAAAAAAAAAAAAAAAAAAAAAAAAAAAAAAAAAA
AAAAAAGTACTCTGCGTTGATACCACTGCTT (70A)

AAGCAGTGGTATCAACGCAGAGTACCGCTGCGAGAGACAGTATGGTGAGCAAGGGCGAGGAGGATAACATGGCCATCATCAAGGAGTTCATGCGCTTCAAGGTGCACATGGAGGGCTCCGTGAACGGCCACGAGTT
CGAGATCGAGGGCGAGGGCGAGGGCCGCCCCTACGAGGGCACCCAGACCGCCAAGCTGAAGGTGACCAAGGGTGGCCCCCTGCCCTTCGCCTGGGACATCCTGTCCCCTCAGTTCATGTACGGCTCCAAGGCCTAC
GTGAAGCACCCCGCCGACATCCCCGACTACTTGAAGCTGTCCTTCCCCGAGGGCTTCAAGTGGGAGCGCGTGATGAACTTCGAGGACGGCGGCGTGGTGACCGTGACCCAGGACTCCTCCCTGCAGGACGGCGAGT
TCATCTACAAGGTGAAGCTGCGCGGCACCAACTTCCCCTCCGACGGCCCCGTAATGCAGAAGAAGACCATGGGCTGGGAGGCCTCCTCCGAGCGGATGTACCCCGAGGACGGCGCCCTGAAGGGCGAGATCAAGCA
GAGGCTGAAGCTGAAGGACGGCGGCCACTACGACGCTGAGGTCAAGACCACCTACAAGGCCAAGAAGCCCGTGCAGCTGCCCGGCGCCTACAACGTCAACATCAAGTTGGACATCACCTCCCACAACGAGGACTAC
ACCATCGTGGAACAGTACGAACGCGCCGAGGGCCGCCACTCCACCGGCGGCATGGACGAGCTGTACAAGTGAAAAAAAAAAAAAAAAAAAAAAAAAAAAAAAAAAAAAAAAAAAAAAAAAAAAAAAAAAAAAAAAAAA
AAAAAAAAAAAAAAAAAAAAAAAAAAAAAAAAAAAAGTACTCTGCGTTGATACCACTGCTT (100A)

b

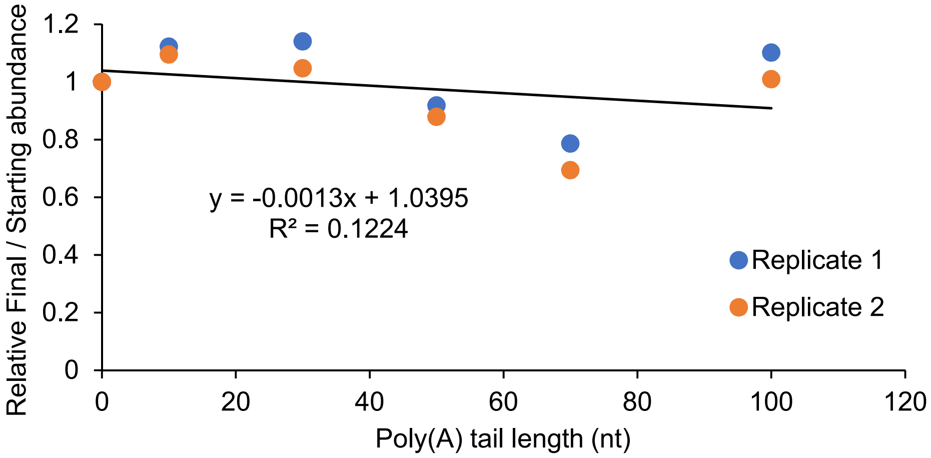

Supplementary Figure 2. The poly(A) spike-ins with 0, 10, 30, 50, 70 and 100As.

(a) The model of the spike-in sequences is shown on the top.

(b) Relative CCS reads recovery rate of the spike-ins of different poly(A) tail length. All spike-ins were normalized to the spike-in of 0 length. The linear regression line is shown in black. The spike-ins are included in two independent runs. Data points for the two replicates are shown in different colors. The slope is -0.0013 and y-axis intercept is 1.0395.

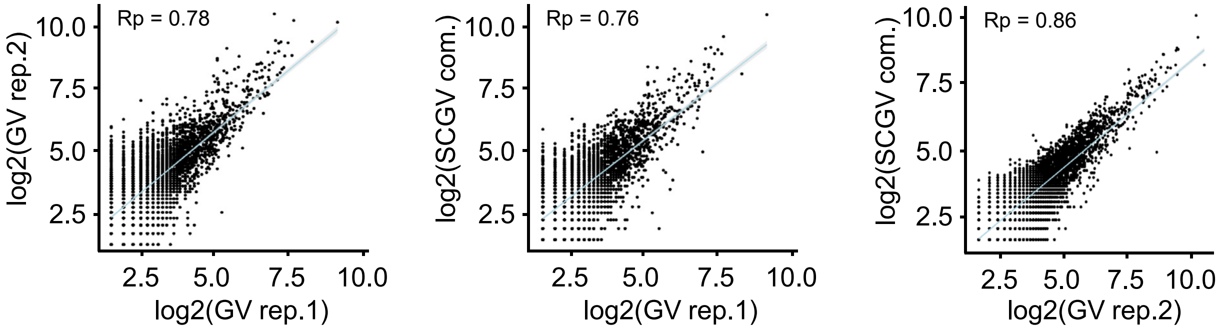

Supplementary Figure 3. The correlation of gene expression among three replicates.

The Pearson correlation of gene expression (transcripts per gene) among three replicates of GV oocytes. GV rep.1: GV replicate1, GV rep.2: GV replicate 2 and SCGV com.: Single cell GV oocytes combined. The blue line represents linear regression line. The light gray area represents confidence interval of the regression. This comparison counts the number of transcript only, therefore, reads used here are not filtered based on passes. n = 4,417 (left panel), n = 4,190 (middle panel), n = 5,258 (right panel).

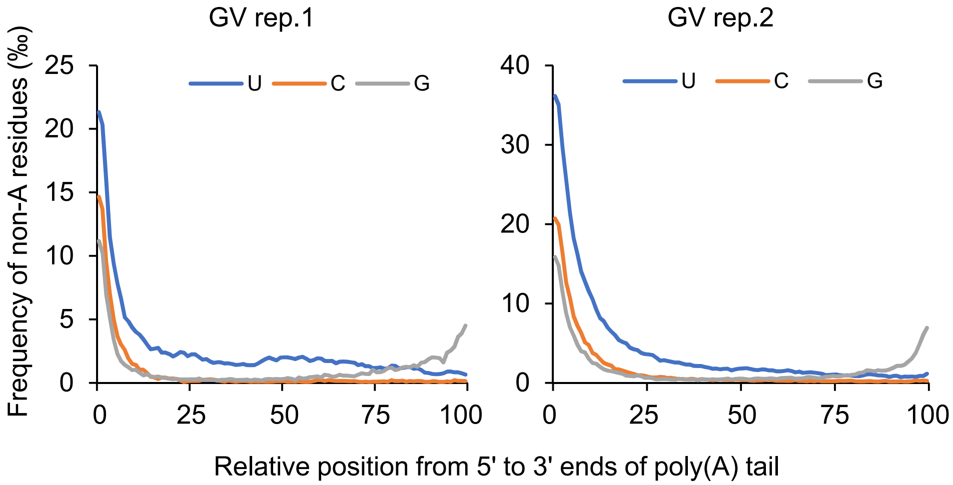

Supplementary Figure 4. Frequency of non-adenosine residues in relative position from 5' to 3' ends of RNA poly(A) tails.

The relative position is from 5' (0) to 3' (100) ends of RNA poly(A) tails. The signal intensity is average of all the poly(A) tails of CCS reads mappable to the genome.

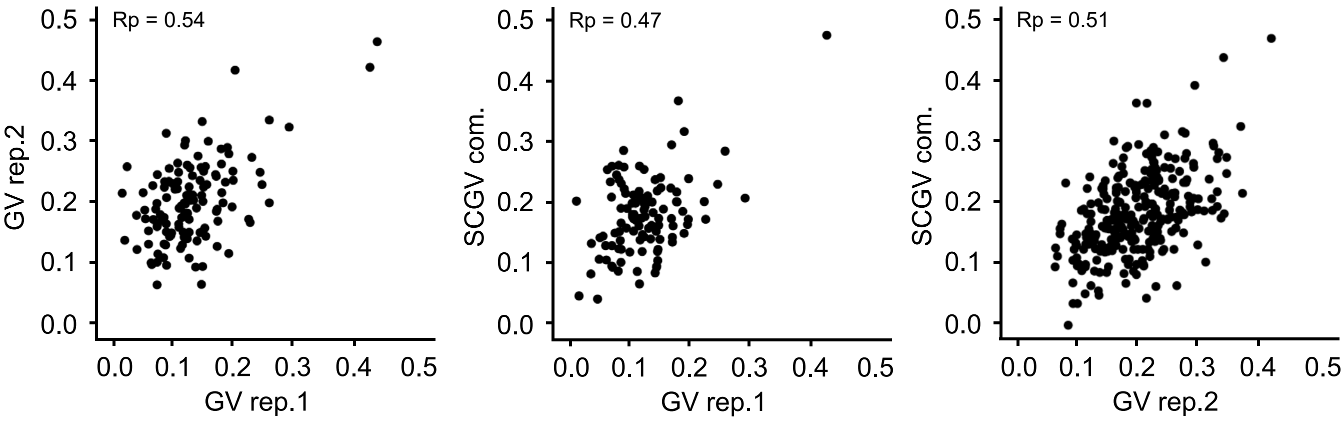

Supplementary Figure 5. Correlation of the ratio of transcripts containing non-A modifications of genes between different replicates of GV oocyte samples.

Genes with no less than 40 CCS reads in each of the replicates are included in the analysis. n = 122 (left panel), n = 108 (middle panel), n = 297 (right panel). “Rp” refers to Pearson correlation coefficient.

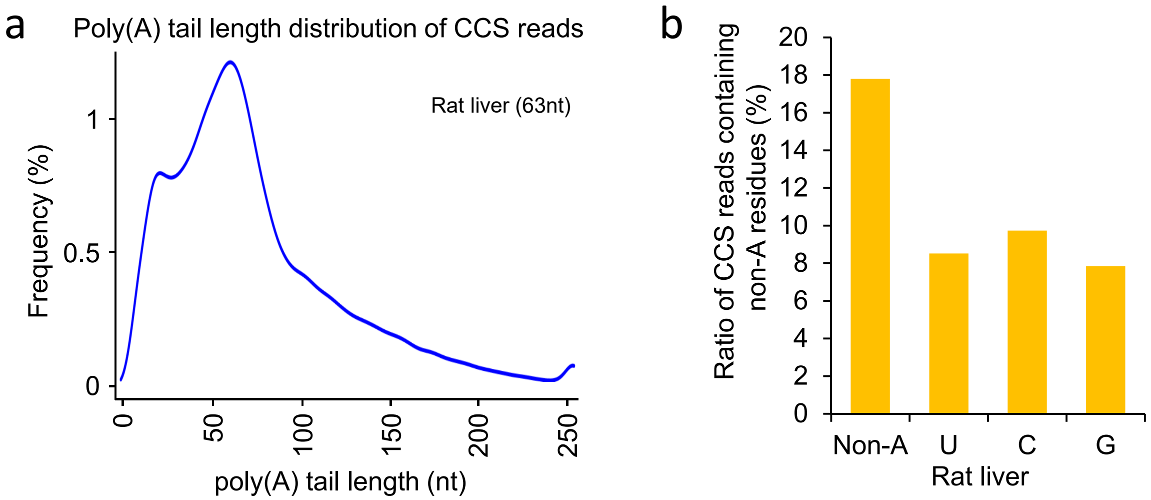

Supplementary Figure 6. Poly(A) tail length and non-A modification ratio in rat liver sample.

(a) The distribution of poly(A) tail length of all genes in rat liver sample. The median poly(A) tail length of the CCS reads is shown on the top.

(b) The ratio of CCS reads containing non-A modifications in rat liver samples.

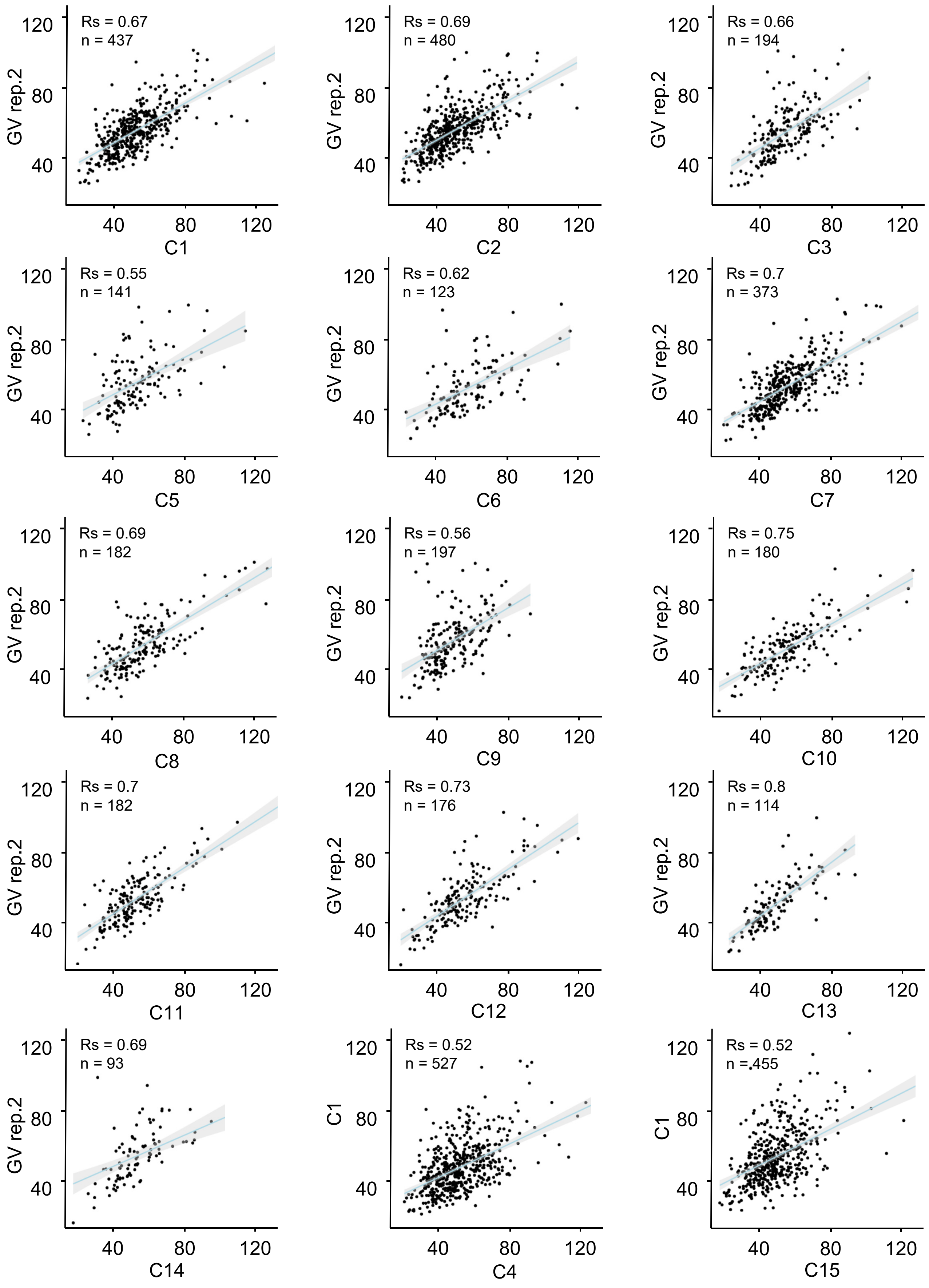

Rs = 0.67
n = 437
GV rep.2
C1
Rs = 0.69
n = 480
GV rep.2
C2
Rs = 0.66
n = 194
GV rep.2
C3
Rs = 0.55
n = 141
GV rep.2
C5
Rs = 0.62
n = 123
GV rep.2
C6
Rs = 0.7
n = 373
GV rep.2
C7
Rs = 0.69
n = 182
GV rep.2
C8
Rs = 0.56
n = 197
GV rep.2
C9
Rs = 0.75
n = 180
GV rep.2
C10
Rs = 0.7
n = 182
GV rep.2
C11
Rs = 0.73
n = 176
GV rep.2
C12
Rs = 0.8
n = 114
GV rep.2
C13
Rs = 0.69
n = 93
GV rep.2
C14
Rs = 0.52
n = 527
C1
C4
Rs = 0.52
n = 455
C1
C15
40
80
120

Supplementary Figure 7. Correlation of poly(A) tail length between single GV oocyte and bulk samples.

C1 (cell 1) to C15 are single-cell samples. GV rep.2 is bulk sample replicate 2. The blue line represents linear regression line. The light gray area represents confidence interval of the regression. Genes with at least 4 CCS reads in each of the datasets are included in the analysis.

Supplementary Table 1. Oligos for PAIso-seq library construction.

**Name**	**Sequence (5'->3')**
TSO	AAGCAGTGGTATCAACGCAGAGTACATrGrG+G
RT primer	AAGCAGTGGTATCAACGCAGAGTAC
PCR primer	AAGCAGTGGTATCAACGCAGAGT
2dU-BC1	AAGCAGTGGTATCAACGCAGAGTACTCAGACGATGCGTCAT dUTTTTTTTTdUTTTTTTTT
2dU-BC2	AAGCAGTGGTATCAACGCAGAGTACCTATACATGACTCTGCd UTTTTTTTTdUTTTTTTTT
2dU-BC3	AAGCAGTGGTATCAACGCAGAGTACTACTAGAGTAGCACTC dUTTTTTTTTdUTTTTTTTT
2dU-BC4	AAGCAGTGGTATCAACGCAGAGTACTGTGTATCAGTACATGd UTTTTTTTTdUTTTTTTTT
2dU-BC5	AAGCAGTGGTATCAACGCAGAGTACGATCTCTACTATATGCd UTTTTTTTTdUTTTTTTTT
2dU-BC6	AAGCAGTGGTATCAACGCAGAGTACACAGTCTATACTGCTGd UTTTTTTTTdUTTTTTTTT
2dU-BC7	AAGCAGTGGTATCAACGCAGAGTAC ATGATGTGCTACATCT dUTTTTTTTTdUTTTTTTTT
2dU-BC8	AAGCAGTGGTATCAACGCAGAGTAC CTGCGTGCTCTACGAC dUTTTTTTTTdUTTTTTTTT
2dU-BC9	AAGCAGTGGTATCAACGCAGAGTAC GCGCGATACGATGACT dUTTTTTTTTdUTTTTTTTT
2dU-BC10	AAGCAGTGGTATCAACGCAGAGTAC CGCGCTCAGCTGATCG dUTTTTTTTTdUTTTTTTTT
2dU-BC11	AAGCAGTGGTATCAACGCAGAGTAC GCGCACGCACTACAGA dUTTTTTTTTdUTTTTTTTT
2dU-BC12	AAGCAGTGGTATCAACGCAGAGTAC ACACTGACGTCGCGAC dUTTTTTTTTdUTTTTTTTT
2dU-BC13	AAGCAGTGGTATCAACGCAGAGTAC CGTCTATATACGTATA dUTTTTTTTTdUTTTTTTTT
2dU-BC14	AAGCAGTGGTATCAACGCAGAGTAC ATAGAGACTCAGAGCT dUTTTTTTTTdUTTTTTTTT
2dU-BC15	AAGCAGTGGTATCAACGCAGAGTAC TAGATGCGAGAGTAGA dUTTTTTTTTdUTTTTTTTT

Note: rG, the ribonucleic guanylic acid; +G, the locked nucleic acid-modified deoxyguanylic acid; dU, the deoxyuridylic acid.

Supplementary Table 2. Oligos for PAT assay.

Name	5'->3'
PAT-C10T2	AAGCAGTGGTATCAACGCAGAGTACCCCCCCCCCTT
PAT-PCR-R	AAGCAGTGGTATCAACGCAGAGT
PAT-Cnot6l-F	TGTGAACATTGACTAAACAAAGCAGTCCTGCC
PAT-Plat-F	ATCCTTGAGATCATGCACTCCCCATCCC
PAT-Zp2-F	ACACAATGACGGTTAGCCTTCCAGGACC
PAT-Npm2-F	CTTAGCATCTTCTGCGTCCAAGGCAGG
PAT-Tle6-F	GACTGAGAACGGCAGGCTCATCATCAC
PAT-Dnmt1-F	GTTTTGTGACCAAGCTGTGTAGTACTTTGTGC
PAT-Btg4-F	GTAGGTTTTCAACTAAGGAAGAT
PAT-Cnot7-F	TGGACTACAAGTTGTAATGTGTG
PAT-Cnot6l-RA0	TGCGGTTGCAAAATGGCCATTTATTGCC
PAT-Plat-RA0	GGAAAAGTGTGAAAAATACCTCTGAATTTATTATTTAAGTACAGTATAATAC
PAT-Zp2-RA0	AACTGCAGTCTCTTTATTTGCAAGTCCAATCAGTG
PAT-Npm2-RA0	CTGATCATGCAAATTTTATTGTGGCCCTAGGCGAG
PAT-Tle6-RA0	GCCATTTCAATTGTTTTATTACACGCAACTCGGG
PAT-Dnmt1-RA0	GGGTGCTTGACAGAAGCGCTTTATTTTGAAG
PAT-Btg4-RA0	GGCTTAGTTTGAATACTTTATTAGAAGCAAGTACCAC
PAT-Cnot7-RA0	ATATAGGAAAACATTTTTGAAGCCTTTATTTACAAAAGCTTTAAAAAC

Supplementary Table 3. Oligos for Poly(A) spike-ins.

PSI-0A-F	AAGCAGTGGTATCAACGCAGAGTACGCACATACACGCTCACATGGTGAGCAAGGGCGAGGAGGATAAC
PSI-0A-R	AAGCAGTGGTATCAACGCAGAGTACTCACTTGTACAGCTCGTCCATGCCGCCGGTG
PSI-10A-F	AAGCAGTGGTATCAACGCAGAGTACGCTCGTCGCGCGCACAATGGTGAGCAAGGGCGAGGAGGATAAC
PSI-10A-R	AAGCAGTGGTATCAACGCAGAGTACTTTTTTTTTTTCACTTGTACAGCTCGTCCATGCCGCCGGTG
PSI-30A-F	AAGCAGTGGTATCAACGCAGAGTACACAGTGCGCTGTCTATATGGTGAGCAAGGGCGAGGAGGATAAC
PSI-30A-R	AAGCAGTGGTATCAACGCAGAGTACTTTTTTTTTTTTTTTTTTTTTTTTTTTTTTTCACTTGTACAGCTCGTCCATGCCGCCGGTG
PSI-50A-F	AAGCAGTGGTATCAACGCAGAGTACTCACACTCTAGAGCGAATGGTGAGCAAGGGCGAGGAGGATAAC
PSI-50A-R	AAGCAGTGGTATCAACGCAGAGTACTTTTTTTTTTTTTTTTTTTTTTTTTTTTTTTTTTTTTTTTTTTTTTTTTTTCACTTGTACAGCTCGTCCATGCCGCCGGTG
PSI-70A-F	AAGCAGTGGTATCAACGCAGAGTACTCACATATGTATACATATGGTGAGCAAGGGCGAGGAGGATAAC
PSI-70A-R	AAGCAGTGGTATCAACGCAGAGTACTTTTTTTTTTTTTTTTTTTTTTTTTTTTTTTTTTTTTTTTTTTTTTTTTTTTTTTTTTTTTTTTTTTTTTTCACTTGTACAGCTCGTCCATGC
PSI-100A-F	AAGCAGTGGTATCAACGCAGAGTACCGCTGCGAGAGACAGTATGGTGAGCAAGGGCGAGGAGGATAAC
PSI-100A-R	AAGCAGTGGTATCAACGCAGAGTACTTTTTTTTTTTTTTTTTTTTTTTTTTTTTTTTTTTTTTTTTTTTTTTTTTTTTTTTTTTTTTTTTTTTTTTTTTTTTTTTTTTTTTTTTTTTTTTTTTTCACTTGTACAGCTCGTCCATGCC

Supplementary Table 4. Genes with transcripts of poly(A) tails longer than 200 nt in all replicates

Replicates	CCS ID	Gene	Passes	Poly(A) tail length (nt)
GV rep.1	m54219_190112_025724/55443787/ccs	Alg13	48	202
GV rep.2	m54308_190404_064332/64422124/ccs	Alg13	81	220
SCGV com.	m54308_190404_170532/59572332/ccs	Alg13	109	207
SCGV com.	m54308_190404_170532/31457392/ccs	Alg13	12	204
GV rep.1	m54219_190112_025724/68551136/ccs	Birc5	26	251
GV rep.2	m54308_190404_064332/24445571/ccs	Birc5	98	213
SCGV com.	m54308_190404_170532/39257071/ccs	Birc5	11	214
GV rep.1	m54219_190125_035319/57738087/ccs	Bpgm	21	244
GV rep.2	m54308_190404_064332/6095376/ccs	Bpgm	24	231
GV rep.2	m54308_190404_064332/54198525/ccs	Bpgm	29	225
GV rep.2	m54308_190404_064332/56165157/ccs	Bpgm	12	203
SCGV com.	m54308_190404_170532/22937765/ccs	Bpgm	11	247
SCGV com.	m54308_190404_170532/50725113/ccs	Bpgm	100	245
SCGV com.	m54308_190404_170532/49545726/ccs	Bpgm	11	202
GV rep.1	m54219_190112_025724/50135571/ccs	Cox6a1	57	221
GV rep.1	m54219_190125_035319/33948208/ccs	Cox6a1	13	209
GV rep.2	m54308_190404_064332/9371713/ccs	Cox6a1	10	301
SCGV com.	m54308_190404_170532/68944188/ccs	Cox6a1	135	204
GV rep.1	m54219_190112_025724/11076152/ccs	Cox6c	26	217
GV rep.2	m54308_190404_064332/48300206/ccs	Cox6c	139	231
GV rep.2	m54308_190404_064332/10617801/ccs	Cox6c	38	218
SCGV com.	m54308_190404_170532/7144372/ccs	Cox6c	14	267
GV rep.1	m54219_190112_025724/61014917/ccs	E330017A01Rik	16	323
GV rep.1	m54219_190112_025724/34406553/ccs	E330017A01Rik	10	244
GV rep.1	m54219_190125_035319/48628617/ccs	E330017A01Rik	21	217
GV rep.1	m54219_190112_025724/71369322/ccs	E330017A01Rik	59	206
GV rep.2	m54308_190404_064332/49152223/ccs	E330017A01Rik	40	331
GV rep.2	m54308_190404_064332/32113109/ccs	E330017A01Rik	25	256
GV rep.2	m54308_190404_064332/6554335/ccs	E330017A01Rik	103	256
GV rep.2	m54308_190404_064332/39911687/ccs	E330017A01Rik	28	255
GV rep.2	m54308_190404_064332/54330289/ccs	E330017A01Rik	35	251

GV rep.2	m54308_190404_064332/5177437/ccs	E330017A01Rik	10	249
GV rep.2	m54308_190404_064332/37617836/ccs	E330017A01Rik	23	235
GV rep.2	m54308_190404_064332/68026482/ccs	E330017A01Rik	15	226
GV rep.2	m54308_190404_064332/38405084/ccs	E330017A01Rik	120	225
GV rep.2	m54308_190404_064332/52363703/ccs	E330017A01Rik	128	204
GV rep.2	m54308_190404_064332/47776172/ccs	E330017A01Rik	98	201
SCGV com.	m54308_190404_170532/42205584/ccs	E330017A01Rik	21	264
GV rep.1	m54219_190112_025724/65995444/ccs	Fbxw14	10	317
GV rep.2	m54308_190404_064332/52035776/ccs	Fbxw14	83	221
GV rep.2	m54308_190404_064332/64291038/ccs	Fbxw14	20	209
SCGV com.	m54308_190404_170532/69665500/ccs	Fbxw14	104	228
SCGV com.	m54308_190404_170532/45940828/ccs	Fbxw14	78	224
SCGV com.	m54308_190404_170532/5701932/ccs	Fbxw14	11	201
GV rep.1	m54219_190112_025724/12779839/ccs	Fbxw24	31	276
GV rep.1	m54219_190125_035319/73532205/ccs	Fbxw24	84	204
GV rep.2	m54308_190404_064332/62652851/ccs	Fbxw24	10	219
GV rep.2	m54308_190404_064332/11797196/ccs	Fbxw24	89	218
GV rep.2	m54308_190404_064332/39322119/ccs	Fbxw24	59	206
SCGV com.	m54308_190404_170532/44761222/ccs	Fbxw24	27	211
GV rep.1	m54219_190112_025724/52232970/ccs	Gdf9	52	240
GV rep.1	m54219_190112_025724/67175334/ccs	Gdf9	66	213
GV rep.2	m54308_190404_064332/35913879/ccs	Gdf9	21	371
GV rep.2	m54308_190404_064332/32310106/ccs	Gdf9	16	322
GV rep.2	m54308_190404_064332/42927002/ccs	Gdf9	81	252
GV rep.2	m54308_190404_064332/43582340/ccs	Gdf9	34	239
GV rep.2	m54308_190404_064332/25822006/ccs	Gdf9	112	226
GV rep.2	m54308_190404_064332/65667638/ccs	Gdf9	30	220
GV rep.2	m54308_190404_064332/5570779/ccs	Gdf9	134	218
GV rep.2	m54308_190404_064332/49348924/ccs	Gdf9	144	216
GV rep.2	m54308_190404_064332/13501044/ccs	Gdf9	27	214
GV rep.2	m54308_190404_064332/66323411/ccs	Gdf9	100	206
SCGV com.	m54308_190404_170532/52953759/ccs	Gdf9	35	240
SCGV com.	m54308_190404_170532/13370184/ccs	Gdf9	48	219
GV rep.1	m54219_190125_035319/5898669/ccs	Ldhb	13	288
GV rep.1	m54219_190112_025724/32440509/ccs	Ldhb	61	236
GV rep.2	m54308_190404_064332/50266919/ccs	Ldhb	88	329

GV rep.2	m54308_190404_064332/15925705/ccs	Ldhb	62	279
GV rep.2	m54308_190404_064332/17105379/ccs	Ldhb	18	270
GV rep.2	m54308_190404_064332/66912748/ccs	Ldhb	21	242
GV rep.2	m54308_190404_064332/34341010/ccs	Ldhb	46	238
GV rep.2	m54308_190404_064332/25363291/ccs	Ldhb	43	233
GV rep.2	m54308_190404_064332/22282679/ccs	Ldhb	15	227
GV rep.2	m54308_190404_064332/36373420/ccs	Ldhb	19	226
GV rep.2	m54308_190404_064332/59113879/ccs	Ldhb	56	226
GV rep.2	m54308_190404_064332/63177576/ccs	Ldhb	16	208
GV rep.2	m54308_190404_064332/59113590/ccs	Ldhb	12	206
SCGV com.	m54308_190404_170532/39322200/ccs	Ldhb	17	251
GV rep.1	m54219_190125_035319/57869099/ccs	Mapk3	56	201
GV rep.2	m54308_190404_064332/51119051/ccs	Mapk3	26	206
SCGV com.	m54308_190404_170532/19202888/ccs	Mapk3	22	235
GV rep.1	m54219_190112_025724/16056482/ccs	Med28	84	238
GV rep.2	m54308_190404_064332/55574854/ccs	Med28	81	214
GV rep.2	m54308_190404_064332/67175318/ccs	Med28	37	201
SCGV com.	m54308_190404_170532/68616890/ccs	Med28	27	206
GV rep.1	m54219_190112_025724/23003401/ccs	Miox	44	218
GV rep.2	m54308_190404_064332/41353580/ccs	Miox	10	298
GV rep.2	m54308_190404_064332/25690280/ccs	Miox	15	235
GV rep.2	m54308_190404_064332/31129753/ccs	Miox	92	205
GV rep.2	m54308_190404_064332/10224624/ccs	Miox	40	204
SCGV com.	m54308_190404_170532/46596811/ccs	Miox	156	224
GV rep.1	m54219_190125_035319/27197886/ccs	Mtmr14	55	219
GV rep.2	m54308_190404_064332/25690449/ccs	Mtmr14	39	213
SCGV com.	m54308_190404_170532/55640349/ccs	Mtmr14	13	221
GV rep.1	m54219_190125_035319/71041117/ccs	Nlrp14	90	218
GV rep.1	m54219_190112_025724/38797930/ccs	Nlrp14	16	202
GV rep.2	m54308_190404_064332/9109731/ccs	Nlrp14	23	279
GV rep.2	m54308_190404_064332/40697921/ccs	Nlrp14	35	254
GV rep.2	m54308_190404_064332/11862771/ccs	Nlrp14	12	219
GV rep.2	m54308_190404_064332/18547284/ccs	Nlrp14	50	216
GV rep.2	m54308_190404_064332/43254456/ccs	Nlrp14	27	212
SCGV com.	m54308_190404_170532/28443479/ccs	Nlrp14	10	231

SCGV com.	m54308_190404_170532/10158148/ccs	Nlrp14	10	221
GV rep.1	m54219_190112_025724/8520010/ccs	Nlrp5	47	217
GV rep.2	m54308_190404_064332/20513089/ccs	Nlrp5	12	333
GV rep.2	m54308_190404_064332/50987544/ccs	Nlrp5	30	227
SCGV com.	m54308_190404_170532/14156647/ccs	Nlrp5	43	266
GV rep.1	m54219_190112_025724/21758246/ccs	Padi6	15	253
GV rep.2	m54308_190404_064332/14811979/ccs	Padi6	75	277
GV rep.2	m54308_190404_064332/36700569/ccs	Padi6	38	275
GV rep.2	m54308_190404_064332/38863593/ccs	Padi6	15	237
GV rep.2	m54308_190404_064332/15074012/ccs	Padi6	35	227
GV rep.2	m54308_190404_064332/32113516/ccs	Padi6	88	226
GV rep.2	m54297_190428_014858/24510676/ccs	Padi6	57	224
GV rep.2	m54308_190404_064332/71958741/ccs	Padi6	114	219
GV rep.2	m54308_190404_064332/74187416/ccs	Padi6	169	215
GV rep.2	m54308_190404_064332/13370065/ccs	Padi6	15	205
GV rep.2	m54308_190404_064332/49283671/ccs	Padi6	104	205
GV rep.2	m54308_190404_064332/21758893/ccs	Padi6	15	204
GV rep.2	m54308_190404_064332/42860640/ccs	Padi6	43	202
SCGV com.	m54308_190404_170532/6554343/ccs	Padi6	46	227
SCGV com.	m54308_190404_170532/20579238/ccs	Padi6	183	214
GV rep.1	m54219_190112_025724/69862371/ccs	Pecr	12	202
GV rep.2	m54308_190404_064332/34996658/ccs	Pecr	40	263
GV rep.2	m54308_190404_064332/48169354/ccs	Pecr	41	247
GV rep.2	m54308_190404_064332/68288651/ccs	Pecr	28	232
GV rep.2	m54308_190404_064332/68485954/ccs	Pecr	33	223
GV rep.2	m54308_190404_064332/35324525/ccs	Pecr	76	216
SCGV com.	m54308_190404_170532/27721893/ccs	Pecr	21	233
GV rep.1	m54219_190112_025724/61800649/ccs	Pttg1	76	243
GV rep.1	m54219_190125_035319/45023411/ccs	Pttg1	94	223
GV rep.1	m54219_190112_025724/12583713/ccs	Pttg1	38	222
GV rep.2	m54308_190404_064332/13238711/ccs	Pttg1	61	313
GV rep.2	m54308_190404_064332/4522918/ccs	Pttg1	55	313
GV rep.2	m54308_190404_064332/19333312/ccs	Pttg1	16	293
GV rep.2	m54308_190404_064332/22479467/ccs	Pttg1	32	241
GV rep.2	m54308_190404_064332/47907010/ccs	Pttg1	63	236
GV rep.2	m54308_190404_064332/36110635/ccs	Pttg1	60	230

GV rep.2	m54308_190404_064332/35717240/ccs	Pttg1	95	227
GV rep.2	m54308_190404_064332/59900714/ccs	Pttg1	55	217
GV rep.2	m54308_190404_064332/16646766/ccs	Pttg1	12	216
GV rep.2	m54308_190404_064332/37422001/ccs	Pttg1	28	211
SCGV com.	m54308_190404_170532/16580979/ccs	Pttg1	12	316
SCGV com.	m54308_190404_170532/60948944/ccs	Pttg1	63	251
SCGV com.	m54308_190404_170532/6423288/ccs	Pttg1	70	240
GV rep.1	m54219_190112_025724/30409052/ccs	Spin1	98	241
GV rep.1	m54219_190112_025724/59965590/ccs	Spin1	15	213
GV rep.1	m54219_190112_025724/30737106/ccs	Spin1	28	203
GV rep.2	m54308_190404_064332/31130225/ccs	Spin1	37	229
GV rep.2	m54308_190404_064332/40764301/ccs	Spin1	60	228
GV rep.2	m54308_190404_064332/21299654/ccs	Spin1	17	219
GV rep.2	m54308_190404_064332/53937104/ccs	Spin1	103	214
SCGV com.	m54308_190404_170532/29885328/ccs	Spin1	94	248
SCGV com.	m54308_190404_170532/45679166/ccs	Spin1	18	243
GV rep.1	m54219_190125_035319/52953633/ccs	Tuba1c	13	241
GV rep.2	m54308_190404_064332/33489086/ccs	Tuba1c	11	210
SCGV com.	m54308_190404_170532/7799689/ccs	Tuba1c	41	201
GV rep.1	m54219_190112_025724/33882516/ccs	Zar1	66	202
GV rep.2	m54308_190404_064332/6030242/ccs	Zar1	18	253
GV rep.2	m54308_190404_064332/43713054/ccs	Zar1	82	240
SCGV com.	m54308_190404_170532/43450540/ccs	Zar1	11	209
GV rep.1	m54219_190112_025724/5112010/ccs	Zp2	43	222
GV rep.1	m54219_190112_025724/66126140/ccs	Zp2	11	206
GV rep.1	m54219_190125_035319/44630411/ccs	Zp2	54	202
GV rep.2	m54308_190404_064332/32572045/ccs	Zp2	48	290
GV rep.2	m54308_190404_064332/37356308/ccs	Zp2	74	218
GV rep.2	m54308_190404_064332/67633674/ccs	Zp2	31	207
SCGV com.	m54308_190404_170532/57082705/ccs	Zp2	75	240
SCGV com.	m54308_190404_170532/24445315/ccs	Zp2	56	213
SCGV com.	m54308_190404_170532/21758810/ccs	Zp2	22	202
SCGV com.	m54308_190404_170532/19268296/ccs	Zp2	17	201
GV rep.1	m54219_190125_035319/20906176/ccs	Zp3	27	214
GV rep.1	m54219_190125_035319/20119773/ccs	Zp3	16	212

GV rep.1	m54219_190112_025724/37093970/ccs	Zp3	100	209
GV rep.2	m54308_190404_064332/37749540/ccs	Zp3	59	296
GV rep.2	m54308_190404_064332/33948390/ccs	Zp3	64	241
GV rep.2	m54308_190404_064332/52757192/ccs	Zp3	114	233
GV rep.2	m54308_190404_064332/66847426/ccs	Zp3	51	220
GV rep.2	m54308_190404_064332/57541248/ccs	Zp3	39	204
SCGV com.	m54308_190404_170532/12779992/ccs	Zp3	34	233
SCGV com.	m54308_190404_170532/50266428/ccs	Zp3	48	218
SCGV com.	m54308_190404_170532/53739588/ccs	Zp3	101	212
SCGV com.	m54308_190404_170532/61014212/ccs	Zp3	86	211
SCGV com.	m54308_190404_170532/45482366/ccs	Zp3	86	210
SCGV com.	m54308_190404_170532/56230467/ccs	Zp3	17	202
