# Supplementary material for: Poly(A) inclusive RNA isoform sequencing (PAIso−seq) reveals wide-spread non-adenosine residues within RNA poly(A) tails
Source: Nat Commun. 2019 Nov 22;10:5292. doi: 10.1038/s41467-019-13228-9 (PMC6876564; doi:10.1038/s41467-019-13228-9)
Supplement: Supplementary file 2 — Reporting Summary [file 41467_2019_13228_MOESM2_ESM.pdf]

nature research

Corresponding author(s): Falong Lu

Last updated by author(s): Oct 19, 2019

Reporting Summary

Nature Research wishes to improve the reproducibility of the work that we publish. This form provides structure for consistency and transparency in reporting. For further information on Nature Research policies, see Authors & Referees and the Editorial Policy Checklist .

Statistics

For all statistical analyses, confirm that the following items are present in the figure legend, table legend, main text, or Methods section.

n/a	Confirmed	
☐	☒	The exact sample size (*n*) for each experimental group/condition, given as a discrete number and unit of measurement
☒	☐	A statement on whether measurements were taken from distinct samples or whether the same sample was measured repeatedly
☐	☒	The statistical test(s) used AND whether they are one- or two-sided<br>*Only common tests should be described solely by name; describe more complex techniques in the Methods section.*
☒	☐	A description of all covariates tested
☒	☐	A description of any assumptions or corrections, such as tests of normality and adjustment for multiple comparisons
☐	☒	A full description of the statistical parameters including central tendency (e.g. means) or other basic estimates (e.g. regression coefficient) AND variation (e.g. standard deviation) or associated estimates of uncertainty (e.g. confidence intervals)
☐	☒	For null hypothesis testing, the test statistic (e.g. *F*, *t*, *r*) with confidence intervals, effect sizes, degrees of freedom and *P* value noted<br>*Give P values as exact values whenever suitable.*
☒	☐	For Bayesian analysis, information on the choice of priors and Markov chain Monte Carlo settings
☒	☐	For hierarchical and complex designs, identification of the appropriate level for tests and full reporting of outcomes
☐	☒	Estimates of effect sizes (e.g. Cohen's *d*, Pearson's *r*), indicating how they were calculated

Our web collection on statistics for biologists contains articles on many of the points above.

Software and code

Policy information about availability of computer code

Data collection	The sequencing data was derived from Pacific BioSciences (PacBio) sequencing platform.
Data analysis	Ccs (version 3.4.1) was used to process subreads generated by PacBio SMRTbell sequencing. Minimap2 (version 2.15-r905) was used to align ccs reads to reference genome. TAPIS (version 1.2.1) was used to detect polyadenylation sites. ClusterProfiler (version 3.10.1) was used for gene set enrichment analysis. R (version 3.5.0) was used to plot violin plot, density plot, bar plot and line plot of PacBio data. Statistical analyses were conducted with R. Key scripts used to process and analyze PAIso-seq data is available on GitHub (https://github.com/niehu2018/GV_oocyte_PAIsoSeqAnalysis).

For manuscripts utilizing custom algorithms or software that are central to the research but not yet described in published literature, software must be made available to editors/reviewers. We strongly encourage code deposition in a community repository (e.g. GitHub). See the Nature Research guidelines for submitting code & software for further information.

Data

Policy information about availability of data

All manuscripts must include a data availability statement. This statement should provide the following information, where applicable:

- Accession codes, unique identifiers, or web links for publicly available datasets
- A list of figures that have associated raw data
- A description of any restrictions on data availability

The PAIso-seq CCS reads have been deposited into NCBI Sequence Read Archive under the accession number PRJNA529588. The SRA accession number of GV rep.1 and GV rep.2 are SRR8798075 and SRR9130368, respectively. The SRA accessions of 15 single GV oocyte PAIso-seq data are SRR9130400, SRR9130401, SRR9130402, SRR9130403, SRR9130404, SRR9130405, SRR9130406, SRR9130407, SRR9130408, SRR9130409, SRR9130410, SRR9130411, SRR9130412, SRR9130413, SRR9130414. All other data are available upon request. The source data underlying Figs 1b and 4d are provided as a Source Data file.

Field-specific reporting

Please select the one below that is the best fit for your research. If you are not sure, read the appropriate sections before making your selection.

- [x] Life sciences
- [ ] Behavioural & social sciences
- [ ] Ecological, evolutionary & environmental sciences

For a reference copy of the document with all sections, see nature.com/documents/nr-reporting-summary-flat.pdf

Life sciences study design

All studies must disclose on these points even when the disclosure is negative.

Sample size	We choosed sample size based on literatures in the field and experimental knowledge.
Data exclusions	No data were excluded from the analyses.
Replication	Almost all experiments shown in this study were performed independently at least three times and no inconsistent results were observed.
Randomization	Samples were randomly distributed into groups.
Blinding	Analysis was objective and did not require blinding.

Reporting for specific materials, systems and methods

We require information from authors about some types of materials, experimental systems and methods used in many studies. Here, indicate whether each material, system or method listed is relevant to your study. If you are not sure if a list item applies to your research, read the appropriate section before selecting a response.

Materials & experimental systems

n/a	Involved in the study	
x		Antibodies
x		Eukaryotic cell lines
x		Palaeontology
	x	Animals and other organisms
x		Human research participants
x		Clinical data

Methods

n/a	Involved in the study	
x		ChIP-seq
x		Flow cytometry
x		MRI-based neuroimaging

Animals and other organisms

Policy information about studies involving animals; ARRIVE guidelines recommended for reporting animal research

Laboratory animals	CD1 (ICR) mice were purchased from Beijing Vital River Laboratory Animal Technology Co., Ltd and bred in our facility. Male and female mice between the age group of 7-8 weeks were used in this study.
Wild animals	The study did not involve wild animals.
Field-collected samples	Field samples were not collected for the study.
Ethics oversight	Mice were maintained in compliance with the guidelines of the Animal Care and Use Committee of the Institute of Genetics and Development Biology, Chinese Academy of Sciences (CAS).

Note that full information on the approval of the study protocol must also be provided in the manuscript.
