# Supplementary material for: Poly(A) inclusive RNA isoform sequencing (PAIso−seq) reveals wide-spread non-adenosine residues within RNA poly(A) tails
Source: Nat Commun. 2019 Nov 22;10:5292. doi: 10.1038/s41467-019-13228-9 (PMC6876564; doi:10.1038/s41467-019-13228-9)
Supplement: Supplementary file 3 — Source Data [file 41467_2019_13228_MOESM3_ESM.pptx]

## Slide 1
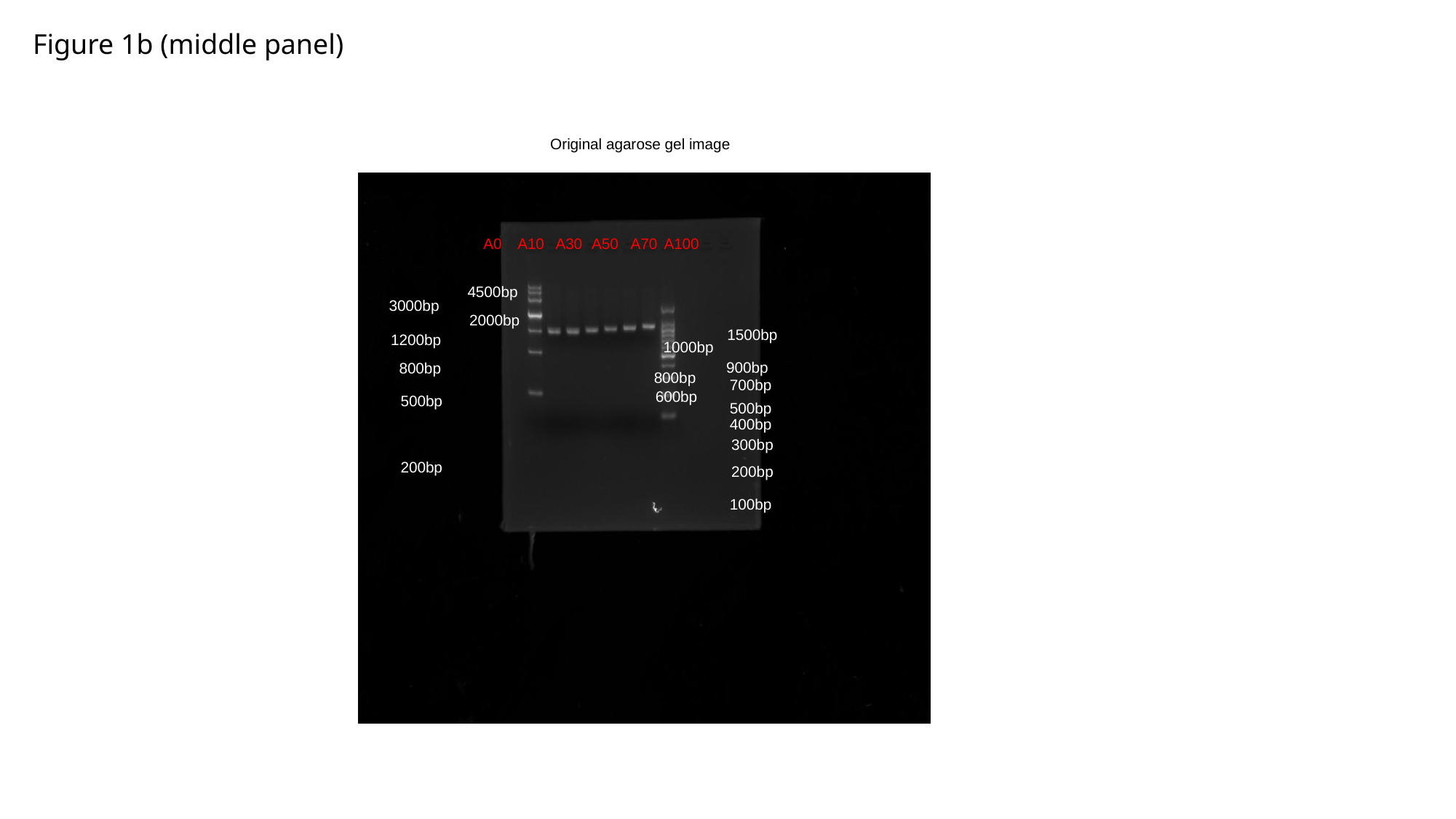

Figure 1b (middle panel)
Original agarose gel image
A0
A10
A30
A50
A70
A100
4500bp
3000bp
2000bp
1500bp
1200bp
1000bp
900bp
800bp
800bp
700bp
600bp
500bp
500bp
400bp
300bp
200bp
200bp
100bp

## Slide 2
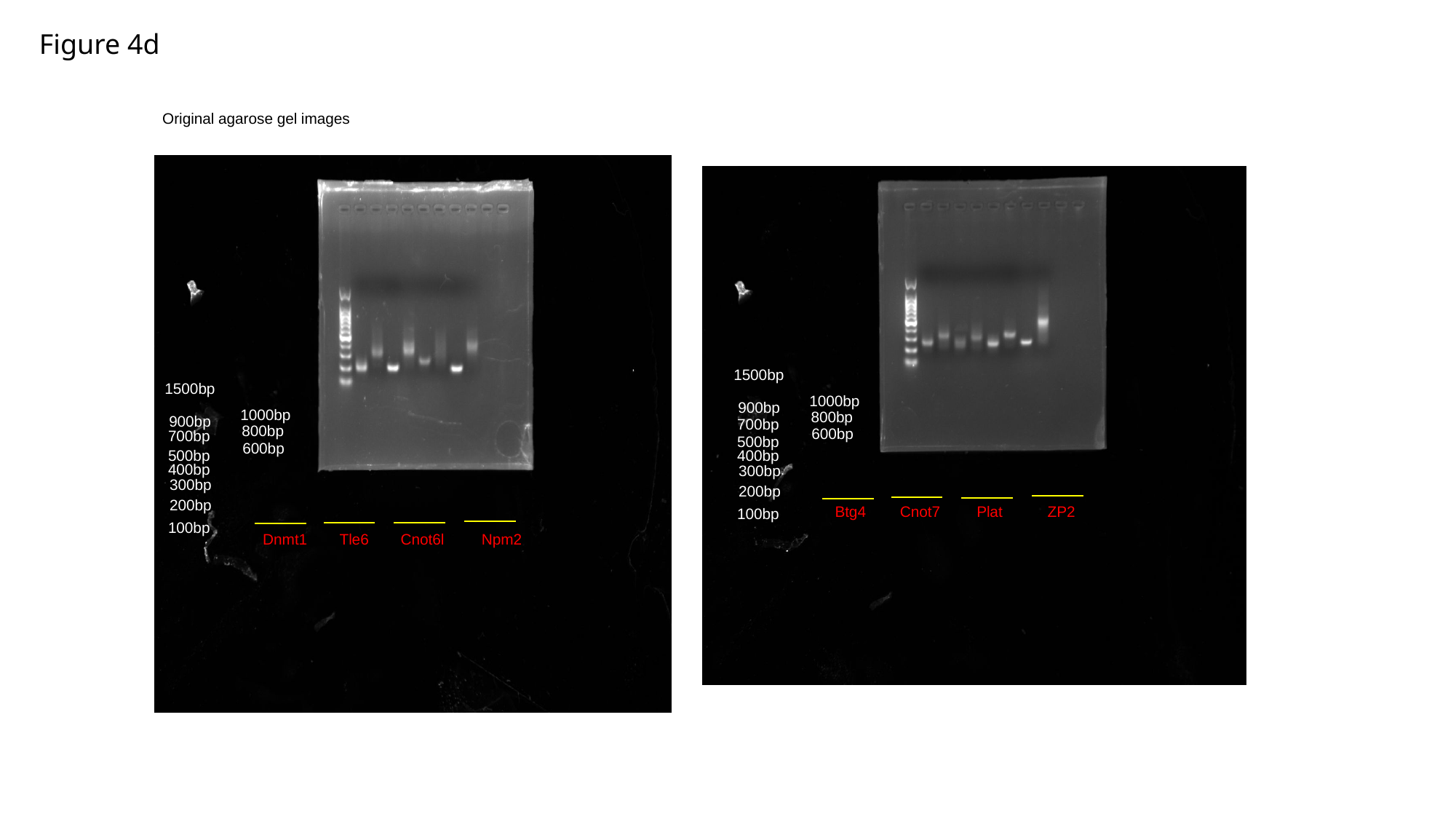

Figure 4d
Original agarose gel images
1500bp
1500bp
1000bp
900bp
1000bp
800bp
900bp
700bp
800bp
600bp
700bp
500bp
600bp
500bp
400bp
400bp
300bp
300bp
200bp
200bp
Btg4
Cnot7
Plat
ZP2
100bp
100bp
Dnmt1
Tle6
Cnot6l
Npm2
